# Mapping non-host resistance to the stem rust pathogen in an interspecific barberry hybrid

**DOI:** 10.1186/s12870-019-1893-9

**Published:** 2019-07-16

**Authors:** Radhika Bartaula, Arthur T. O. Melo, Sarah Kingan, Yue Jin, Iago Hale

**Affiliations:** 1 Department of Molecular, Cellular, and Biomedical Sciences, University of New Hampshire, Durham, NH 03824 USA; 2 Department of Agriculture, Nutrition, and Food Systems, University of New Hampshire, Durham, NH 03824 USA; 3 Pacific Biosciences, Menlo Park, CA 94025 USA; 4 USDA-ARS Cereal Disease Laboratory, St. Paul, MN 55108 USA

**Keywords:** Wheat, Stem rust, Barberry, Non-host resistance, Durable resistance, Reference genome

## Abstract

**Background:**

Non-host resistance (NHR) presents a compelling long-term plant protection strategy for global food security, yet the genetic basis of NHR remains poorly understood. For many diseases, including stem rust of wheat [causal organism *Puccinia graminis* (*Pg*)], NHR is largely unexplored due to the inherent challenge of developing a genetically tractable system within which the resistance segregates. The present study turns to the pathogen’s alternate host, barberry (*Berberis* spp.), to overcome this challenge.

**Results:**

In this study, an interspecific mapping population derived from a cross between *Pg*-resistant *Berberis thunbergii* (*Bt*) and *Pg*-susceptible *B. vulgaris* was developed to investigate the *Pg*-NHR exhibited by *Bt.* To facilitate QTL analysis and subsequent trait dissection, the first genetic linkage maps for the two parental species were constructed and a chromosome-scale reference genome for *Bt* was assembled (PacBio + Hi-C). QTL analysis resulted in the identification of a single 13 cM region (~ 5.1 Mbp spanning 13 physical contigs) on the short arm of *Bt* chromosome 3. Differential gene expression analysis, combined with sequence variation analysis between the two parental species, led to the prioritization of several candidate genes within the QTL region, some of which belong to gene families previously implicated in disease resistance.

**Conclusions:**

Foundational genetic and genomic resources developed for *Berberis* spp. enabled the identification and annotation of a QTL associated with *Pg*-NHR. Although subsequent validation and fine mapping studies are needed, this study demonstrates the feasibility of and lays the groundwork for dissecting *Pg*-NHR in the alternate host of one of agriculture’s most devastating pathogens.

**Electronic supplementary material:**

The online version of this article (10.1186/s12870-019-1893-9) contains supplementary material, which is available to authorized users.

## Background

Stem rust, caused by the fungal pathogen *Puccinia graminis* (*Pg*), has for millennia been one of the most destructive diseases of wheat and related small grains [1–3]. Effective control of the disease was realized in the middle of the twentieth century through the concerted development of resistant wheat varieties and the removal of *Pg*’s alternate host, common barberry (*Berberis vulgaris* L.), from major wheat growing areas [3, 4]. In the last 20 years, however, the emergence of new virulent stem rust races has rendered some long-used resistance genes ineffective [5, 6]. For example, when the wheat stem rust race *Ug99* was first detected in East Africa in 1998, more than 80% of the world’s wheat germplasm was estimated to be vulnerable to its unprecedented virulence on the widely-deployed resistance gene *Sr31* [7]. The rapid distribution and continued evolution of the *Ug99* family of races, combined with recent stem rust outbreaks in Europe [8], underscore the need for new sources of resistance [9]. Traditionally, such new sources have been sought almost entirely from within the diverse *Triticum* genepool. Although translatability to wheat improvement may be less straightforward, or potentially even unachievable, a complementary approach may to look beyond this genepool for potential mechanisms of non-host resistance (NHR) to the complex *Pg* pathogen.

NHR is a form of resistance in which all individuals of a potential host species exhibit immunity to all individuals (e.g. races) of a potential pathogen [10]. As the most common form of disease resistance and one that possesses intrinsic durability, NHR presents a compelling strategy for achieving broad-spectrum, durable protection against many plant pathogens, including the causal organism of wheat stem rust [11, 12]. The genetic mechanisms underlying *Pg*-NHR remain largely unknown, especially in comparison to the relatively well-studied mechanisms of race specific and quantitative, race non-specific host resistance. Over the past decade, however, efforts have mounted to understand NHR to rust pathogens using various model and non-model plants. Many plant species, including *Arabidopsis thaliana*, *Brachypodium distachyon*, rice, barley, and cowpea [13–18], have been used to study NHR to *P. striiformis* f. sp. *tritici*, the causal organism of wheat stripe rust. In contrast, NHR to the wheat stem rust pathogen *Pg* has thus far been studied only in rice [13], as distinct from the studies of intermediate *Pg* resistance conducted in barley and *B. distachyon* [19, 20].

As the only globally important small grain immune to all known rust diseases, rice (*Oryza* spp.) presents a logical potential source of *Pg*-NHR genes. Genetic studies of *Pg*-NHR in rice are difficult, however, precisely because populations of non-hosts fail, by definition, to segregate for resistance. Although some limited progression of *Pg* infection has been shown in rice, thus raising the possibility of dissecting *Pg*-NHR in that system, the infection process exhibits little variation, requires tedious microscopic studies to characterize, and ultimately fails to complete [13]. As an alternative to rice, the *Berberis*-*Pg* system was recently proposed as a tractable pathosystem for studying the genetics of *Pg*-NHR [21]. Numerous species within the highly diverse *Berberis*, or barberry, genus are susceptible to *Pg* infection (e.g. European barberry *B. vulgaris* L., the target of massive eradication efforts from wheat-growing regions in the twentieth century) [22, 23]. Others, however, are considered non-hosts. Japanese barberry *B. thunbergii* DC., for example, is considered a non-host of *Pg* due to two lines of evidence: 1) Over nearly a century of extensive testing at the USDA Cereal Disease Lab, no *Pg* infection has ever been observed in the species [24–33], and 2) No *Pg* infection has been observed on *B. thunbergii* under natural conditions, despite rampant proliferation of the species in the landscape. Because hybridization between such host and non-host species are known to occur in nature (e.g. *B.* ×*ottawensis* C.K. Scheid) [34], populations of interspecific barberry hybrids present a potential means of mapping and dissecting the genetic basis of *Pg*-NHR.

**Fig. 1:**
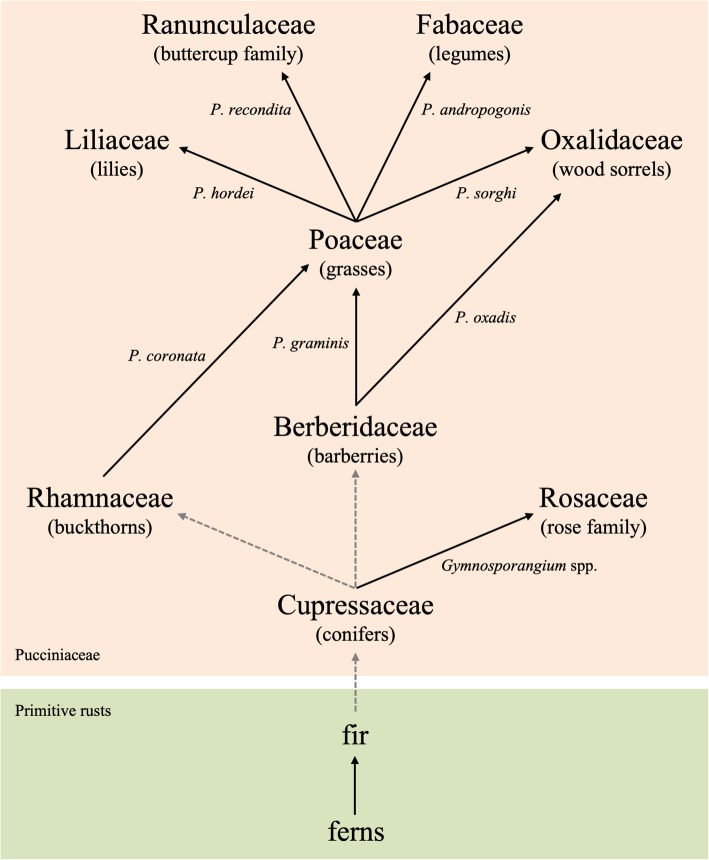
Schematic of the hypothesized evolution of modern day macrocyclic, heteroecious *Puccinia* species**.** Host jump is believed to be a frequent event in the evolution of rusts, and barberries are thought to be one of the first eudicots parasitized by the rusts. Dotted arrows indicate a lack of existing rust species connecting the respective host groups. In such cases, connection is indirectly supported by relative morphological similarity among teliospores. Solid arrows connect two host species between which the indicated rust species alternate

The barberries are a compelling model for other reasons as well. Unlike rice, which has no known co-evolutionary relationship with *Pg*, barberries are thought to be one of the first eudicots parasitized by the rusts (Fig. 1). Indeed, multiple lines of evidence support the idea that the barberries may have played an important role in the evolution of the rust fungi. First, *Berberis* spp. host a wide diversity of rusts, including numerous macrocyclic, heteroecious species of *Puccinia* (e.g. *Pg, P. striiformis, P. montanensis, P. brachypodii, P. pigmea, P. koeleriae*, and *P. arrhenatheri*), a number of autoecious rusts (e.g. *Cumminsiella* spp., belonging to Pucciniaceae; *Edythea* spp., belonging to Uropyxidaceae; and *Pucciniosira* spp., belonging to Pucciniosiraceae), and even some anamorphic rusts (e.g. *Acedidium* and *Uredo* spp.). Second, only slight morphological differences exist among the teliospores of the various macrocyclic rusts [35], suggesting a single evolutionary origin of these pathogens. Third, a recent palaeobotanical finding of *B. wuyunensis* from a sediment layer between 55 to 65 million years ago in northeastern China suggests that the barberries are one of the earliest groups of angiosperms [36].

More specific to the grass rusts, there are eight known *Puccinia* spp. that complete their sexual (aecial) stage on barberry and their asexual (uredinial and telial) stages on graminaceous plants from the Poaceae family. This relationship, in combination with the relative ages of these two plant families, suggests that *Puccinia* spp. likely parasitized the Berberidaceae prior to their host expansion to the grasses. Today, the genus *Puccinia* is comprised of more than 2000 species; and within that diverse genus, host jump rather than co-speciation is believed to be the primary means of speciation [37]. As more recent examples, a host jump from Poaceae to Ranunculaceae likely produced the *P. recondita* complex and its aligned species, a jump to Liliaceae likely produced *P. hordei* and its aligned species, and a jump to Oxalidaceae likely produced *P. sorghi* and its aligned species. Because the relationship between barberries and the rusts likely predates such speciation (Fig. 1), it is of fundamental interest to probe the mechanism(s) of NHR exhibited by some contemporary species of barberry.

In this study, an interspecific *B.* ×*ottawensis* mapping population was created to study the inheritance of the gene(s) underlying the putative *Pg-*NHR of *B. thunbergii*. To support this work, necessary genetic and genomic resources were developed, including genetic linkage maps for the two parental species (*B. thunbergii* and *B. vulgaris*) and a chromosome-scale reference genome for *B. thunbergii*. This study not only establishes foundational resources for the *Berberis*-*Pg* pathosystem but also demonstrates their use in an initial dissection of *Pg*-NHR, with the long-term hope of contributing insight into possible novel mechanisms of durable resistance to the stem rust pathogen.

## Results

### Variant detection and linkage map construction

Genotyping-by-sequencing (GBS) libraries were constructed for the two parental lines (*B. vulgaris* accession ‘Wagon Hill’ and *B. thunbergii* accession ‘BtUCONN1’) and their 182 interspecific *B.* ×*ottawensis* F_1_ progeny, generating a total of 60 Gb of data [~ 401 million 150-bp paired end (PE) reads]. After quality parsing and demultiplexing, an average of 3 million high quality reads per genotype were retained by the GBS-SNP-CROP pipeline [38] (Additional file 1). Using the high quality reads from the two parents, a mock reference (MR) comprised of 87,089 centroids (i.e. consensus GBS fragments) was generated, comprising a total length of approximately 15.4 Mbp.

A total of 15,411 polymorphic markers, including 14,043 SNPs (average depth D_SNPs_ = 41.5) and 1368 indels (D_indels_ = 36.4), were identified by mapping all high-quality reads from the population to the MR. A detailed account of the winnowing of these markers via a progression of filters to obtain the final sets of markers for linkage map construction is provided in Table 1. Separate genetic linkage maps were constructed for each parental species, using a two-way pseudo-testcross mapping strategy [39]. After culling individual F_1_ progeny with > 30% missing data, 161 and 162 individuals were retained for *B. thunbergii* and *B. vulgaris* linkage map construction, respectively. The *B. thunbergii* map was constructed using a total of 1757 markers (1497 and 260 from Marker Sets 1 and 2, respectively; see Table 1), and the *B. vulgaris* map was constructed using a total of 706 markers (600 and 106 from Marker Sets 3 and 4, respectively). For both parental species, the remaining markers coalesced into 14 distinct linkage groups, in agreement with the reported chromosomal number in these *Berberis* spp. (Additional file 2: Figure S1).

**Table 1:** Description of the sequence of filters applied to obtain the final marker sets for linkage map construction

Filter descriptions, in order of application	Markers removed at each step	Markers retained
1. More than 30% missing genotype calls across the population^a^	6106	9305
2. Heterozygous in both parents	272	9033
3. Homozygous for alternate alleles in the two parents^b^	3982	5051
4. Deviates significantly from expected allele depth ratio in heterozygotes^c^	1801	3250
5. Segregating genotypes unsupported by parental genotypes^d^	697	2553
6. Deviates significantly from expected Mendelian segregation^e^	90	2463
Final markers for the *B. thunbergii* linkage map (*Bt* × *Bv*)		1757
Marker Set 1: ab × aa		1497
Marker Set 2: cd × −-		260
Final markers for the *B. vulgaris* linkage map (*Bt* × *Bv*)		706
Marker Set 3: ee × ef		600
Marker Set 4: -- × gh		106

^a^ This first filter was applied to the initial set of 15,411 markers (SNPs and indels) identified by the GBS-SNP-CROP pipeline

^b^ If both parents are homozygous for the marker, no variation will be observable among the progeny (i.e. all F_1_ progeny will be heterozygous for the marker)

^c^ Mean allele depth ratio across heterozygous F_1_ progeny deviates > 25% from the expected bi-allelic depth ratio of 1:1

^d^ Lack of parental genotypes (missing data) and/or parental genotyping errors can prevent the unique assignment of gametic origin. For example, while ab × aa is expected to segregate only as aa and ab among the progeny, the alternate homozygote (bb) may be observed due to parental genotyping error. All such markers were removed from the analysis

^e^ Segregation ratio of genotypes deviates more than two standard deviations from the expectation for each marker set; such markers were removed due to their high segregation distortion

Summary statistics of the two genetic linkage maps are detailed in Table 2. The *B. thunbergii* map consists of 598 recombination bins (i.e. mapped loci) and has a total length of 1474 cM. The numbers of bins in each of the 14 linkage groups (LGs) range from 23 (LG14) to 60 (LG2), with an average distance between adjacent bins of 2.6 cM. In comparison, the *B. vulgaris* map consists of 347 bins and a total length of 1714 cM. The numbers of bins in each of these 14 LGs range from 13 (LG14) to 37 (LG2), with an average distance between adjacent bins of 5.5 cM. Marker names, alleles, and genetic positions (cM), as well as a color-coded visualization of the recombination events within all members of the mapping population are provided in Additional file 3 (*B. thunbergii*) and Additional file 4 (*B. vulgaris*).

**Table 2:** Comparative summary statistics of the genetic linkage maps for *B. thunbergii* accession ‘BtUCONN1’ (*Bt*) and *B. vulgaris* accession ‘Wagon Hill’ (*Bv*)

Linkage Group^a^	Length (cM)		Number of markers		Number of loci		Mean distance between loci (cM)	
	*Bt*	*Bv*	*Bt*	*Bv*	*Bt*	*Bv*	*Bt*	*Bv*
1	122.1	145.5	178	74	60	37	2.1	4.0
2	98.1	130.4	122	63	41	31	2.5	4.3
3	119.7	134.1	140	58	50	28	2.4	5.0
4	110.8	154.1	139	44	48	28	2.4	5.7
5	101.6	143.0	109	54	47	27	2.2	5.5
6	115.7	135.0	195	76	49	31	2.4	4.5
7	112.0	121.4	151	33	50	21	2.3	6.1
8	102.0	120.2	145	44	34	26	3.1	4.8
9	96.8	134.6	148	63	46	32	2.2	4.3
10	93.0	121.2	88	25	34	14	2.8	9.3
11	87.4	64.6	110	39	43	21	2.1	3.2
12	118.3	86.8	75	35	39	15	3.1	6.2
13	101.0	139.7	100	52	34	23	3.1	6.3
14	95.9	83.3	55	32	23	13	4.4	6.9
Average	105.3	122.4	125.4	49.4	42.7	24.8	2.6	5.5

^a^ Linkage group designations (1–14) are based on the *B. thunbergii* cv. ‘Kobold’ genome assembly. Linkage Group 1 anchors to the longest pseudo-molecule in the Kobold assembly (99.76 Mbp); Linkage Group 2 to the next longest (99.56 Mbp); as so on to Linkage Group 14 (54.72 Mbp) (see Additional file 2: Table S2)

### Disease phenotyping

**Fig. 2:**
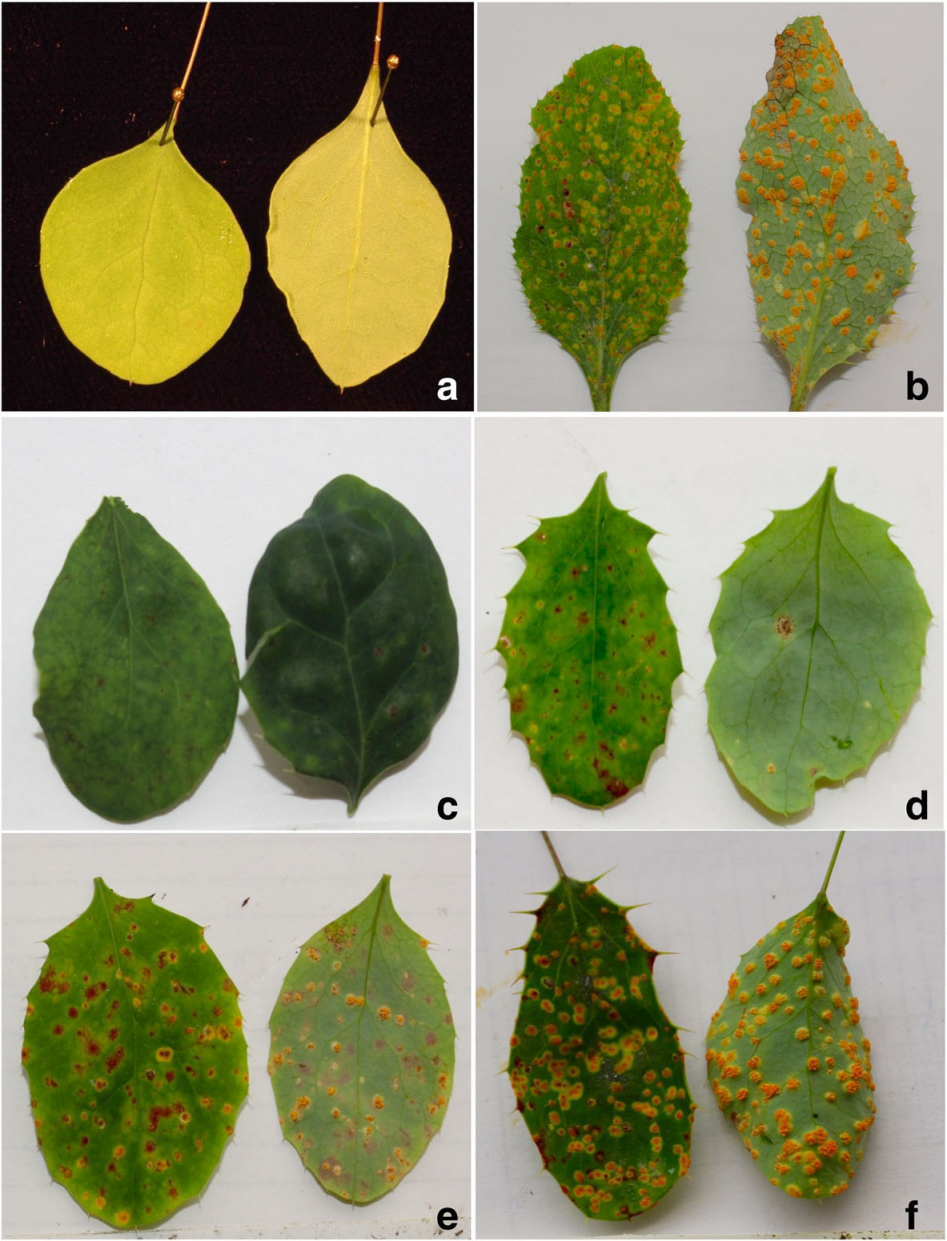
Representative disease responses of the two mapping population parents and their F_1_ progeny. **a** Resistant reaction of *B. thunbergii* accession ‘BtUCONN1’, showing no visual symptoms; **b** Susceptible reaction of *B. vulgaris* accession ‘Wagon Hill’, showing dense pycnia on the upper leaf surface and prolific, well-developed aecia on the lower surface; **c** Resistant reaction (score of 1 on the four-point scale) of *B.* ×*ottawensis* progeny ‘WH15–039’, showing sparse flecking; **d** Moderate resistant reaction (score 2) of *B.* ×*ottawensis* progeny ‘WH15–063’, showing evident necrotic lesions and some pycnia formation; **e** Moderate susceptible reaction (score 3) of *B.* ×*ottawensis* progeny ‘WH15–128’, showing well-developed pycnia and aecia, alongside sparse necrotic lesions; and **f** Susceptible reaction (score 4) of *B.* ×*ottawensis* progeny ‘WH15–149’, showing well-developed pycnia and aecia and no evident necrosis. All photos were taken 14 days post-inoculation

To determine disease responses to *Pg*, the parents and all F_1_ progeny were inoculated with basidiospores ejected from germinated teliospores produced by overwintered telia of *Pg* found on naturally infected *Elymus repens*. The progeny segregated into four clear phenotypic classes, ranging from resistant to susceptible (Fig. 2, Table 3). Disease phenotypes were successfully obtained for 153 progeny used for linkage map construction. Of those, 25 exhibited a clear resistant reaction similar to that of the *B. thunbergii* parent (Fig. 2c) and 61 exhibited a clear susceptible reaction similar to that of the *B. vulgaris* parent (Fig. 2f). Of the remaining 67 lines, 38 exhibited moderate resistance (Fig. 2d) and 29 exhibited moderate susceptibility (Fig. 2e).

**Table 3:** Descriptions of the disease reactions of the *B.* ×*ottawensis* progeny comprising the F_1_ mapping population

Scale^a^	Description
1	Sparse flecking and necrotic lesions, up to 3 tiny pycnia without aecia on a leaf
2	Evident necrotic lesions; 5 to 15 obvious pycina with or without aecia on a leaf
3	5 to 15 well developed pycnia with aecia on a leaf; sparse necrotic lesions
4	> 15 well-developed pycnia and aecia; not preceded by necrosis

^a^ A general 4-point scale was developed for phenotyping the disease reactions of the F_1_ mapping population. A resistant (R) reaction is indicated by a score of 1, a moderate resistant (MR) reaction by 2, a moderate susceptible (MS) reaction by 3, and a susceptible (S) reaction by 4. Representative images for the four disease classes are shown in Fig. 2

### QTL analysis

**Fig. 3:**
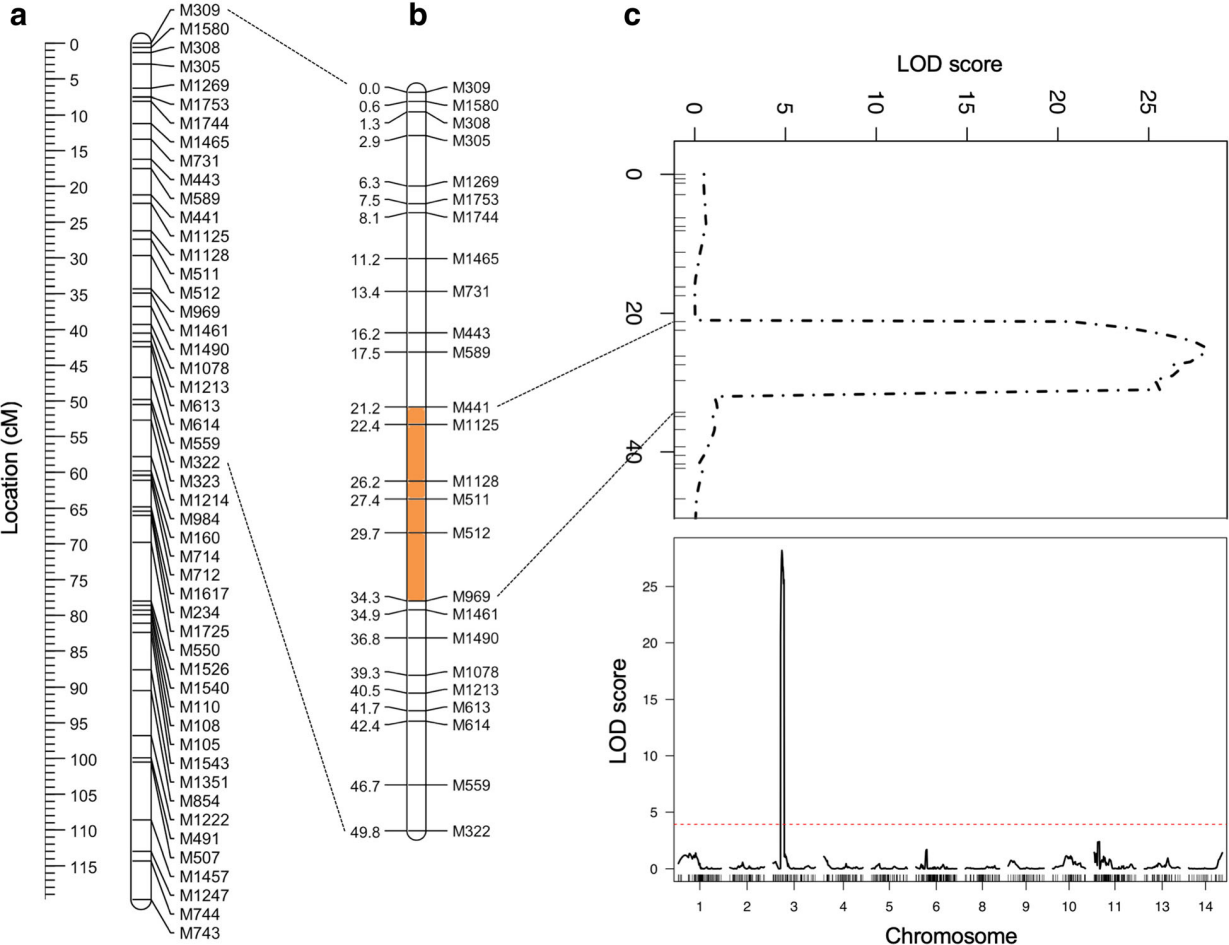
The *QPgr-3S* region on the short arm of *B. thunbergii* chromosome 3. **a** Full genetic linkage map of chromosome 3; **b** Enlarged linkage map of the short arm of chromosome 3, with the *QPgr-3S* QTL region indicated in orange between its two flanking markers, *M411* and *M969*; **c** LOD plot of the *QPgr-3S* region (top) and the context of the single QTL peak across the 14 chromosomes of *B. thunbergii* (bottom). The dotted red line indicates the threshold for QTL significance (LOD = 3.9), determined via permutation analysis

To map regions associated with *Pg*-NHR in *B. thunbergii*, composite interval mapping (CIM) analysis was conducted using the linkage maps of both parents and the 4-point stem rust reaction type described above. Based on the LOD threshold score of 3.9 declared via permutation analysis, CIM analysis resulted in the identification of a single significant QTL (peak LOD value = 28.2) located 25 cM from the telomere of the short arm of *B. thunbergii* chromosome 3 (Fig. 3). The flanking markers for this 13 cM QTL region, hereafter referred to as *QPgr-3S*, were determined via a detailed characterizations of the F_1_ individuals with recombination events on either side of peak QTL marker *M1128*. The distal flanking marker *M441* is set by *Pg*-resistant individual WH15–192, and the proximal flanking marker *M969* is set by *Pg*-resistant individual WH15–101 (Additional file 3). No significant QTL was detected in the *B. vulgaris* map*.*

### Building a reference genome for *B. thunbergii* cv. ‘Kobold’

Approximately 129 Gb of sequence data was generated from 115 PacBio Single Molecule Real Time (SMRT) cells (P6-C4 chemistry on RS II), with an average read length of 10,409 bp and a read length N50 of 15,021 bp (Additional file 2: Table S1). The haploid genome size of Kobold, a widespread green-leafed *B. thunbergii* ornamental cultivar, was estimated to be 1.37 Gbp based on k-mer analysis and 1.72 Gb based on flow cytometry (data not shown), two values which bound the previously published *B. thunbergii* haploid genome size (1C) of 1.51 Gb [40]. The FALCON-Unzip pipeline [41] resulted in a 1.36 Gb assembly consisting of 4671 primary contigs with contig length N50 of 0.67 Mbp (Table 4). Their corresponding 7144 phased haplotigs had a total length of 0.88 Gb, approximately 64% of the primary contig space. Further curation, in the form of chimera breaking and cryptic haplotig identification (see Materials and Methods), resulted in a final 1.23 Gbp assembly consisting of 2698 primary contigs with contig length N50 of 0.76 Mbp (Table 4). The number of haplotigs in the final assembly increased to 8790, with a combined length of 0.99 Gb (> 80% of the primary contig space).

**Table 4:** Summary statistics of the *B. thunbergii* cv. ‘Kobold’ genome assembly, by stage

Variables	FALCON-Unzip		Final assembly^a^		Hi-C scaffolding^b^
	Primary contigs	Haplotigs	Primary contigs	Haplotigs	
Number of contigs	4671	7144	2698	8790	14
Total length (Gbp)	1.36	0.88	1.23	0.99	1.20
Longest (Mbp)	8.60	1.49	8.60	1.49	99.76
Shortest (bp)	8581	561	20,469	561	54.72
> 100 kbp (%)	2551 (54.6)	2836 (39.7)	2229 (82.6)	3126 (35.6)	14 (100)
> 1 Mbp (%)	289 (6.2)	9 (0.1)	289 (10.7)	9 (0.1)	14 (100)
Mean length (Mbp)	0.29	0.12	0.46	0.11	85.40
N50 length (Mbp)	0.67	0.21	0.76	0.19	88.62
GC content (%)	37.6	37.7	37.7	37.7	37.66

^a^ After application of the Purge Haplotigs pipeline [61] and manual contig curation (i.e. chimera breaking and haplotig re-assignment)

^b^ All statistics for the Hi-C assembly refer to scaffolds rather than contigs. For details of individual pseudo-molecules, see Additional file 2: Table S2

Genome completeness and contamination analyses revealed a final genome assembly of acceptable quality, featuring complete representation of 80.9% of the BUSCO core plant gene set and only 15.1% missing BUSCO genes. 83.0% of the BtUCONN1 GBS fragments, 80.71% of the PacBio preads, and 92.2% of the RNA-seq data (in proper pair) aligned to the final assembly. After the initial FALCON-Unzip assembly, 119 primary contigs showed significant sequence similarity to plant cpDNA and mtDNA sequence; but this number dropped to only one primary contig in the final assembly as a result of intensive haplotig purging and curation.

The primary contigs from the final assembly were guided into chromosome-level scaffolds (pseudo-molecules) on the basis of three-dimensional proximity information obtained via chromosome conformation capture analysis (Hi-C) [42]. Of the 2698 primary contigs, 97% (2611 contigs, 1.20 Gbp) successfully assembled into 14 pseudo-molecules representing the 14 chromosomes of *B. thunbergii*, as shown in the Hi-C heatmap (Additional file 2: Figure S2). The remaining 3% (156 contigs, 33.5 Mbp) were designated as unscaffolded contigs. Detailed summary statistics of the 14 pseudo-molecules comprising the *B. thunbergii* cv. ‘Kobold’ reference assembly can be found in Additional file 2: Table S2.

### Anchoring the genetic linkage maps to the physical assembly and assigning chromosome numbers

**Fig. 4:**
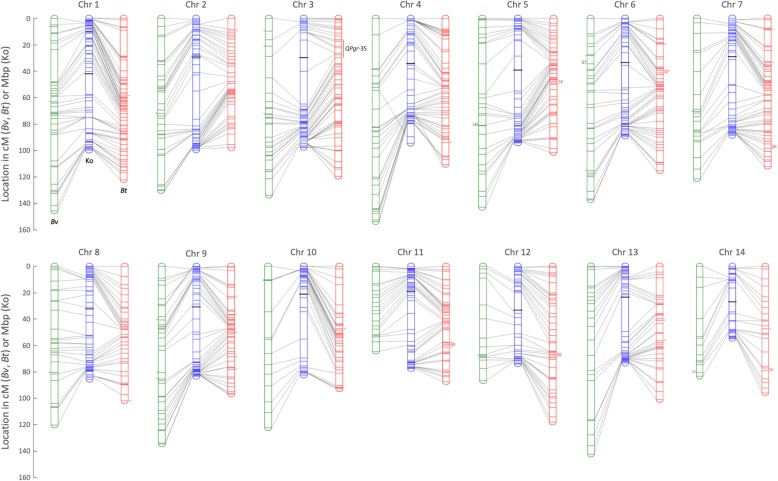
Visualization of synteny of the BtUCONN1 (red) and Wagon Hill (green) genetic maps. *Berberis vulgaris* (*Bv*, green) and *B. thunbergii* (*Bt*, red) genetic maps (in cM) are anchored to the *B. thunbergii* cv. ‘Kobold’ reference assembly (Ko, blue; in Mbp) via GBS centroids. The seven GBS markers that BLAST outside their expected linkage groups are indicated by small numbers (01–14) that signify the linkage groups with which they associate. The four GBS markers that BLAST to unscaffolded contigs are indicated by “Un”. Small dots beside linkage maps indicate loci with multiple, ambiguous alignments throughout the genome. Bold horizontal black bars on the Kobold physical map indicate approximate centromere positions, based on the Hi-C heatmap. The position of QTL region *QPgr*-3S is indicated alongside the chromosome 3 linkage map for the *B. thunbergii* parent ‘BtUCONN1’

Using BLASTn with MR centroids as queries, the positions of the mapped GBS markers within the final Hi-C assembly were used to anchor the genetic linkage maps of both parental species to the Kobold physical map. As illustrated in Fig. 4, a very high degree of synteny is observed between the two species, with co-linearity to the Kobold physical map being 95.1 and 92.9% for the *B. thunbergii* and *B. vulgaris* linkage maps, respectively. The physical positions of a small percentage of loci in both linkage maps (3.9% in *B. thunbergii* and 5.1% in *B. vulgaris*) were ambiguous, in that they could not be assigned to unique positions in the physical assembly. Another small percentage of loci (0.93% in *B. thunbergii* and 1.12% in *B. vulgaris*) exhibited unambiguous BLAST hits to different chromosomes than in the linkage map, as indicated by dots in Fig. 4. The approximate centromere positions were visually inferred from the Hi-C heatmap (Additional file 2: Figure S2).

To assign chromosome numbers to linkage groups, the pseudo-molecules from the Kobold physical assembly were sorted, longest to shortest. The linkage group (LG) that anchored to the longest pseudo-molecule in the Kobold assembly (99.76 Mbp) was designated LG1; the next longest pseudo-molecule was designated LG2 (99.56 Mbp); and so on to LG14 (54.72 Mbp) (see Additional file 2: Table S2). Because there was perfect agreement between the number of observed linkage groups and the expected chromosome number for the species [40], LG1 was simply re-assigned as Chromosome 1 and so on.

### Transcriptome assembly

A total of 59.6 Gb of data, comprised of ~ 198 million 150-bp PE reads, was obtained by sequencing a library of 10 different tissues from the *B. thunbergii* reference accession ‘Kobold’, including immature leaf tissues sampled at various time points following inoculation with *Pg* (Additional file 2: Table S3). Using the Trinity pipeline [43] and the final Kobold assembly as a guide, a 189.3 Mbp transcriptome was assembled, containing 122,872 putative transcripts and 55,186 cDNA sequences (complete ORFs) (see Table 5 for summary statistics). Quality and completeness of the transcriptome assembly were assessed via TransRate [44] and BUSCO analysis [45]. To date, a TransRate score of 0.22 exceeds 50% of the published de novo assembled transcriptomes deposited in the NCBI TSA [44]. In comparison, the TransRate score of the Kobold transcriptome is 0.40, indicating its relative quality. Completeness statistics are also acceptable, as indicated by the fact that, of the BUSCO set of 1440 core plant genes, 1286 (89.3%) were represented in the transcriptome, of which 651 (45.2%) were single copy and 635 (44.1%) were duplicated.

**Table 5:** Descriptive statistics of the *B. thunbergii* cv. ‘Kobold’ reference-guided transcriptome assembly

Trinity reference-based assembly results	
Number of transcripts	122,872
Total length (bp)	189,291,041
Mean length (bp)	1541
Number of ORFs (%)	55,186 (44.28%)
Transcript length N50 (bp)	1991
GC Content	40.0%
TransRate results	
TransRate score	0.403
TransRate optimal score	0.427
TransRate cutoff	0.037
Number of good contigs (%)	120,972 (98.5%)
BUSCO results	
Complete (%)	1286 (89.3%)
Complete and single-copy (%)	651 (45.2%)
Complete and duplicated (%)	635 (44.1%)
Fragmented (%)	47 (3.3%)
Missing (%)	107 (7.4%)

### Identification of candidate genes

The 13 cM *QPgr-3S* region was found to correspond to a 5.35 Mbp region in the physical assembly, implicating 20 contigs (length N50 = 389.7 kbp). In an effort to refine the assembly within the QTL region, these 20 contigs were locally re-assembled using canu [46], resulting in a final set of 13 contigs with a reduced total length of 5.10 Mbp and an increased contig length N50 of 508.5 kbp. Using RepeatMasker [47], 5.6% (~ 373 kbp) of the *Qpgr-3S* region was masked as repetitive elements using *A. thaliana* as the model. A total of 219 retroelements were found, of which 178 are LTRs (79 *Ty1*/*Copia* and 99 *Gypsy*/*DIRS1*) and 41 are LINEs (*L1*/*CIN4*). Another approximately 9 kbp of sequence were found to correspond to DNA transposons. Regions of simple sequence repeats occupy a total length of 130 kbp, and 32 small RNAs were found.

Functional annotation of the *QPgr-3S* region resulted in the identification of 576 high confidence (HC) genes. Of these, 450 were annotated based on the reference transcriptome (evidence-based) and 126 were annotated based on gene prediction models (ab initio). To help identify a short list of candidate genes potentially associated with *Pg*-NHR and prioritized for ongoing investigation, the list of HC genes was cross-referenced to the results of two other analyses: Differential gene expression (DGE) and presence/absence analysis (see Materials and Methods). Time course DGE analysis led to the identification of five genes (TR27614, TR9306, TR20791, TR5393, and TR12856) that express differentially under *Pg* inoculation (Additional file 2: Figures S3 and S4). Genes TR27614 and TR9306 exhibit a similar pattern of gradual down-regulation starting around 48 h post-inoculation (hpi). Gene TR20791 exhibits up-regulation during the first 48 hpi, followed by down regulation after 72 hpi. In contrast, genes TR5339 and TR12856 appear initially down-regulated before gradually climbing back to their original levels after 72 hpi. Presence/absence analysis identified two genes that are present in the *B. thunbergii* reference but appear to be either completely absent (MA26) or are missing whole exons (MA262) in *B. vulgaris* (Additional file 2: Figure S5). The evidence for possible absence in *B. vulgaris* is particularly strong with MA026 due to the high coverage of *B. vulgaris* reads in the immediate vicinity of the gene (Additional file 2: Figure S5).

Combined with the linkage evidence from the QTL analysis, the results of the time course DGE and presence/absence analyses elevate the seven genes identified above to a status of candidate genes associated with *Pg*-NHR. As such, these candidates were selected for detailed functional annotation; and orthologous sequences were found for three of them (TR20791, TR27614, and TR12856) in the UNIPROT and Phytozome databases. Specifically, gene TR20791 is associated with a dormancy-related auxin repressor protein family; TR27614 exhibits high sequence similarity with zinc finger DNA-binding proteins; and TR12856 belongs to the glutamine synthetase (glutamate-ammonia ligase activity) protein family (Additional file 5). The other four candidate genes had no hits in any public database used for functional annotation and thus are potentially *Berberis*-specific genes, or at the very least are novel genes previously uncharacterized in other species. As the application of next-generation sequencing has become routine in genomic studies, identification of high numbers of completely novel transcripts has been found to be common in both model and non-model species (e.g. see [48–51]).

## Discussion

### Genetic and genomic resource development

Familiar, commonly used mapping populations for genetic linkage map construction in plants include segregating F_2_ lines, backcross populations, doubled haploids, and recombinant inbred lines. In self-incompatible perennial plant species, however, particularly those with long generation times like barberries, such typical mapping populations are difficult, if not impossible, to produce. To overcome such challenges, the so-called “pseudo-testcross” strategy was first proposed by Grattapaglia and Sederoff (1994) and successfully applied to construct a genetic linkage map in forest trees [39]. According to this strategy, a mapping population of full-sib F_1_ progeny is developed by crossing two unrelated and highly heterozygous (i.e. not inbred) individuals. Gametic recombinations can be tracked in such a population because strategically-chosen sets of markers obey the segregation patterns found in typical testcrosses. The strategy has been widely used in plant species for which other approaches are unsuitable [52–54].

In this study, using a pseudo-testcross strategy, genetic linkage maps were developed for both *B. thunbergii* and *B. vulgaris* from a single interspecific F_1_ mapping population. As a result of the stringent quality filters applied to the set of de novo GBS markers used, nearly 100% of the markers were placed successfully in the linkage maps of the two species. Although flow cytometry analysis indicates comparable genome sizes between the two parents (*B. thunbergii*: 1.72 Gbp; *B. vulgaris*: 1.69 Gbp), the total length of the BtUCONN1 (*B. thunbergii*) linkage map obtained in this study is roughly 15% smaller than that of the Wagon Hill (*B. vulgaris*) map (1474 cM vs. 1714 cM). This incongruity with the expected differences in physical genome sizes is likely due to the significantly fewer markers available for the *B. vulgaris* map as compared to those available for *B. thunbergii* (706 vs. 1757)*.* Low marker density often results in inflated genetic distances [55], so it is expected that additional markers would reduce the overall length of *B. vulgaris* linkage map. The significantly lower number of markers available for *B. vulgaris* is likely a result of the relatively lower level of diversity observed in this species as a result of the severe genetic bottleneck presumed during its colonial introduction from Europe into North America [21].

The two linkage maps developed in this study are the first for any species within the plant order Ranunculales. The relatively even distribution of markers across the 14 chromosomes of both species permits initial QTL analysis of acceptable resolution, with approximately 87 and 65% of the inter-marker distances being less than 5 cM for *B. thunbergii* and *B. vulgaris*, respectively. In addition, the strong synteny observed between the two independent maps is strong evidence of their reliability (Fig. 4).

As a complement to genetic resources like mapping populations and linkage maps, a high-quality reference genome can serve as an invaluable resource in dissecting QTLs, identifying underlying candidate genes, and facilitating their detailed characterization. In this study, contemporary sequencing and scaffolding technologies were used to develop a highly contiguous de novo reference genome for *B. thunbergii*. Using PacBio SMRT sequencing and chromosome conformation capture data, a 1.2 Gb haploid assembly of *B. thunbergii* cv. ‘Kobold’ was successfully assembled into 14 chromosome-scale pseudo-molecules. As with the linkage maps, this reference is the first of its kind for a member of both the Berberidaceae family as well as the order Ranunculales, more broadly. Given the previous lack of molecular resources for barberries, the reference genome assembled in this study exemplifies the power of recent technologies to make rapid progress even in non-model systems and establishes a benchmark for the de novo assembly of a highly heterozygous plant species with a moderately sized genome.

In conclusion, the development of foundational genetic and genomic resources, including a genotyped interspecific mapping population, linkage maps for its two parental species, a chromosome-scale reference genome, and a multiple-tissue transcriptome establishes *Berberis* spp. as a viable research model for studying *Pg*-NHR. Furthermore, such resources promise to facilitate related endeavors, including global rust surveillance work and ornamental horticulture breeding.

### *QPgr*-*3S* and the identification of candidate genes for *Pg*-NHR

The long-term goal of this research is to identify candidate gene(s) governing *Pg*-NHR in *B. thunbergii*. As an initial step in that direction, the genetic and genomic resources developed here enabled the identification of a single QTL of large effect (LOD > 28) on the short arm of *B. thunbergii* chromosome 3 (Fig. 3). This 13 cM QTL region, dubbed *Qpgr-3S*, was found to span 13 physical contigs and contain a total of 576 high-confidence genes. Of these, seven were short-listed as relatively high priority candidate genes for follow up studies, including three exhibiting homology to genes in public databases, including dormancy-associated auxin repressor proteins (TR20791), zinc ion binding proteins (TR27614), and glutamine synthetase proteins (TR12856).

The current model of disease resistance suggests that plant immune responses can be grouped broadly into two major classes, namely pre-invasion defense triggered by pathogen-associated molecular patterns (PAMP-triggered immunity) and post-invasion defense triggered by pathogen effectors (effector-triggered immunity) [56, 57], both of which have been shown to implicate a wide range of defense-related proteins. Three of the seven candidate genes identified here in this study exhibit homology to gene families implicated in disease resistance in the literature. For example, auxin is known to function as a modulator of salicylic acid, a phyto-hormone essential to the induction of systemic acquired resistance in plants [58]; zinc finger transcription factors have been implicated in the regulation of a gene affecting rust germ tube differentiation [59]; and glutamine synthetase proteins are known to play key roles in plant defense against pathogens via amino acid metabolism [60].

The identification of both the *QPgr-3S* region and a set of high-priority candidate genes demonstrates the utility of the genetic and genomic resources developed in the study to probe the genes underlying *Pg*-NHR exhibited by *B. thunbergii*. Such results, however, are but the first step toward identifying the genes governing *Pg*-NHR; and further work is required to validate and dissect the QTL region, in addition to testing candidate gene hypotheses.

### Possible modes of inheritance of *Pg*-NHR

From the practical standpoint of breeding for improved resistance to wheat stem rust, the central questions regarding *Pg*-NHR concern the nature and modes of inheritance of the underlying genes. As previously observed in a natural interspecific barberry hybrid population [21], F_1_ interspecific hybrids exhibit a range of reactions to *Pg*, from fully resistant to fully susceptible, with various intermediate forms. This range of reactions was similarly observed in the F_1_ mapping population developed for this study (Fig. 2c-f and Table 3). If one assumes that the *Pg*-resistance in *B. thunbergii* is governed by a single gene, independent assortment during meioses would invariably result in homozygous *Pg*-susceptible *B. thunbergii* progeny. To date, however, no accession of *B. thunbergii* has exhibited such susceptibility, despite extensive investigation (see Background); thus a single gene governing the *Pg*-resistance in *B. thunbergii* is unlikely. Polygenic NHR has been suggested in other studies as well, including rice NHR to wheat stem rust and barley NHR to powdery mildews, oat stem rust, and other non-adapted rust species [19, 61, 62].

If indeed the *QPgr-3S* region plays a role in *Pg*-NHR, the data suggest that its underlying gene(s) are necessary but not sufficient for resistance. In other words, this study at most provides a first insight into a larger gene network regulating *Pg*-NHR in *B. thunbergii*. Indeed, in light of the lack of segregation in the non-host parental species *B. thunbergii*, the segregation of resistance among F_1_ hybrids suggests the possible existence of some critical gene(s), by definition fixed within the *B. thunbergii* genepool, upstream of *QPgr-3S*. Because of their fixed state within *B. thunbergii*, such gene(s) cannot be mapped in an F_1_ population; but if recessive, their single dosage in an F_1_ would permit susceptibility to *Pg*, thus allowing the detection of background resistance genes (e.g. *QPgr-3S*). In all likelihood, *QPgr-3S* is not a critical region conferring *Pg*-NHR but is rather a region contributing to *Pg* resistance. Strategic crosses among the F_1_ progeny and/or backcrosses to *B. thunbergii* will be necessary to test this hypothesis and identify those critical gene(s) regulating *Pg*-NHR in *B. thunbergii*, work shown to be feasible by the current study.

## Conclusions

In this paper, we report the development of publicly-available foundational genetic and genomic resources for the novel *Berberis*-*Pg* pathosystem, including the first genetic maps for two *Berberis* species (*B. thunbergii* and *B. vulgaris*), a chromosome-scale reference genome for *B. thunbergii*, and a related transcriptome to facilitate the characterization of genetic mechanism(s) of *Pg*-NHR. Future work should focus on the validation, further characterization, and dissection of the identified QTL, including testing of candidate gene hypotheses. Beyond this, now that the *Berberis*-*Pg* pathosystem has been shown to be a viable means of probing the mechanism of *Pg*-NHR in *B. thunbergii*, future work must also wrestle with the significant question of potential translatability of such resistance to wheat. Such translatability is certainly not a given, particularly in light of the fact that the infecting spores are different for *Berberis* (basidiospores) and grass (urediniospores) hosts. However, because the two life stages in question belong to the same pathogenic organism and because *Berberis* is the likely ancestral host of that organism prior to its host expansion to the grasses (see Background), the possibility exists that the mechanism of *Pg*-NHR in *B. thunbergii* may provide relevant insight into breeding durable resistance in wheat. With this study, the foundation is laid to eventually answer this question.

## Methods

### Mapping population development

A *B.* ×*ottawensis* mapping population consisting of 182 F_1_ individuals was derived from an interspecific cross between *B. thunbergii* accession ‘BtUCONN1’ (pollen parent) and *B. vulgaris* accession ‘Wagon Hill’ (female parent). True to its species, BtUCONN1 is a non-host to the stem rust pathogen and is a small shrub (0.5–2.5 m tall) that displays 1.3–3.8 cm long entire leaves and 1–2 cm long inflorescences with few umbellate but mostly solitary flowers. In contrast, Wagon Hill is susceptible to stem rust and is a relatively taller shrub (~ 3 m tall) that displays 2–5 cm long obovate to obovate-oblong leaves with highly serrated margins (> 50 serrations) and has 5–8 cm long pendant racemes of bright yellow flowers. The pollen parent BtUCONN1 was a feral plant maintained in the barberry collection at the research farm of the University of Connecticut (N41°47′40.63″, W072°13′39.61″), and the female parent Wagon Hill is a feral plant growing along the shoreline of the Great Bay Estuary in Durham, New Hampshire (N43°07′30.64″, W70°52′17.95″).

To make the interspecific cross, pollen was harvested from mature flowers of BtUCONN1 using the previously described N-pentane method [63] and stored at 4 °C until flowers of Wagon Hill reached reproductive maturity. Emasculation and hand pollination of female flowers were performed at the so-called balloon stage, when the petals begin to part slightly at the top, giving the appearance of an inflated balloon prior to opening. To break dormancy before sowing, seeds from successful crosses were stratified in wet sand in a petri dish at 4 °C for three months. Propagated cuttings of the two parents were maintained along with the F_1_ mapping population in plastic pots (11.5 cm diameter; 6.5 cm tall) filled with PRO-MIX HP growth media in the Macfarlane Greenhouse facility at the University of New Hampshire.

To verify the putative F_1_ status of the individuals in the mapping population, a PCR-based species-specific marker was designed based on available GBS data [21]. A universal primer pair was designed to amplify a short genomic sequence exhibiting a length polymorphism between the two parents. Specifically, the primers (F: 5′-CCTGATTGGGGCTCATTATC-3′; R: 5′-AGTGAGGAATTCCGAGCTGA-3′) amplified a 208 bp fragment in Wagon Hill but only a 195 bp fragment in BtUCONN1, due to the presence of a 13 bp indel (see Additional file 6: Text S1). PCR was conducted with a total reaction volume of 20 μl (0.25 mM of each primer, 100 μM of each dNTP, 0.75 U Taq DNA Polymerase, 10x standard Taq buffer, and 100 ng of template DNA) subjected to the following cycling conditions: 5 mins at 94 °C; 32 cycles of 30 s at 94 °C, 30 s at 52 °C, and 15 s at 68 °C; and 5 mins at 68 °C. Amplified products were separated on a 3% TBE/EtBr agarose gel for 60 min at 75 V and imaged with UV transillumination. The F_1_ status of a putative hybrid individual was considered validated if both bands from the two parental species were detected (Additional file 2: Figure S6).

### Genotyping and variant detection

Genomic DNA of the 182 verified F_1_ individuals and both parents was extracted from ~ 100 mg of lyophilized leaf tissue using a modified CTAB method [64]. Prior to GBS library preparation, isolated DNA was purified using Zymo Research’s Genomic DNA Clean & Concentrator™^-10^ column (Catalog # D4011), following manufacturer’s protocol. Reduced representation libraries were constructed using the two-enzyme (*PstI-MspI)* GBS protocol described by Poland et al. [65] and sequenced via 150 bp paired-end (PE) reads on an Illumina HiSeq 2500 at the Hubbard Center for Genome Studies, UNH.

Raw FASTQ files were generated by CASAVA 1.8.3 and analyzed using the reference-free bioinformatics pipeline GBS-SNP-CROP [38, 66]. A Mock Reference (MR) was constructed using the high quality PE reads from the two parents; and putative variants, both SNPs and indels, were identified via alignment of high quality PE reads from the parents and all F_1_ progeny to the MR, following the pipeline’s recommended parameters for diploid species. Complete details of the GBS-SNP-CROP command lines used in this analysis, including all specified pipeline parameters, are provided in Additional file 6: Text S2.

### Genetic linkage map construction

The sequence of filters applied to obtain the final sets of markers for linkage map construction is summarized in Table 1. In short, a marker was culled if it met any of the following criteria: 1) It was unscored for more than 30% of the individuals in the population; 2) It was heterozygous for both parents; 3) It failed to segregate in the population (i.e. all progeny were heterozygous for the marker); 4) Its mean ratio of primary to alternate allele depth deviated significantly from the expected ratio of 1:1; and/or 5) Its segregation ratio deviated significantly from the expected ratio of 1:1, according to its marker class. As a final filter, genotypes with > 30% missing data were removed.

Linkage analysis was performed using the R package ONEMAP v2.0–4 [67], and separate linkage maps were constructed for the two parents according to a two-way pseudo-testcross mapping strategy [30]. The BtUCONN1 linkage map was constructed using Marker Sets 1 and 2, while the Wagon Hill map was constructed using Marker Sets 3 and 4 (see Table 1). For each map, a two-point test was first performed for all marker pairs, using a minimum LOD score of 4 and a maximum recombination fraction of 0.25 to group markers into linkage groups (LGs). Next, markers within each LG were ordered using the ‘try’ algorithm within ONEMAP.

To identify potential genotyping errors, common in GBS data [68], maps were manually inspected for the presence of singletons (apparent double crossovers) [69], which were replaced with missing values. If multiple markers were found to map to the same genetic bin, a consensus of the set of markers was chosen to represent the linkage bin for final mapping iterations, which were made until no alternative orders were generated by the ‘ripple.seq’ function. Final map distances were calculated with the Kosambi mapping function [70], and ideograms were generated using Mapchart 2.0 [71].

### Stem rust disease phenotyping

To determine disease responses, the parents and all F_1_ individuals in the mapping population were inoculated with basidiospores ejected from germinated teliospores produced by *Pg* telia found on naturally-infected *Elymus repens*, as previously described [21]. The pollen parent BtUCONN1 exhibits the clear non-host reaction typical of *B. thunbergii*. In contrast, the female parent Wagon Hill exhibits the clear susceptible reaction of *B. vulgaris*, with well-developed mature aecia visible on the abaxial surfaces of leaves. Images of typical reactions of the parents and of individuals in the F_1_ mapping population are presented in Fig. 2. As detailed in Table 3, a 4-point scale was developed in response to the particular segregating characteristics observed in this population. The levels of this scale are based on the following symptoms: 1) Degree of flecking; 2) Presence and intensity of necrotic lesions; and 3) Presence and density of pycnia and aecia. All plants were scored for reaction to stem rust 14 days after inoculation.

### QTL analysis

QTL analysis for *Pg* resistance was performed using both the parental and maternal genetic linkage maps using the R package R/qtl v1.39–5 [72]. Haley-Knott regression [73] was used, based on the composite interval mapping method (CIM); and a QTL was deemed significant if its peak LOD score exceeded the threshold determined via permutation analysis (1000 permutations, 5% significance level).

### Reference genome assembly

Due to its relevance not only to *Pg*-NHR research but also to ornamental breeding, *B. thunbergii* cv ‘Kobold’, a commercial green-leafed cultivar common in the ornamental industry, was selected for whole genome sequencing. Kobold is a heterozygous diploid (2n = 2x = 28) and is a non-host to stem rust [33]. Cuttings of Kobold were obtained from the barberry collection at the University of Connecticut, rooted, and maintained in the MacFarlane Greenhouses at UNH under standard conditions for barberry [21]. For sequencing, ~ 2 g of fresh young leaves were collected from 4 to 6 clonally propagated plants and flash frozen in liquid nitrogen. Genomic DNA was extracted using a modified CTAB procedure [74] and quantified via both fluorometry (Qubit, Thermo Fisher Scientific, Waltham, U.S.A.) and agarose gel electrophoresis with a lambda DNA standard. A 20-kb BluePippin kit (PacBio) was used for Single Molecule Real Time (SMRT) library preparation; and 115 SMRT cells were sequenced on the PacBio RS II system at the UC Davis Genome Center, using P6-C4 chemistry. All data were collected as 6­h sequencing videos.

The FALCON and FALCON-Unzip toolkits (FALCON-integrate v1.8.2) [41] were used for whole genome assembly and phasing. FALCON is described as a Hierarchical Genome Assembly Process pipeline that generates a genome assembly from long PacBio reads through the following basic steps: 1) Raw read error correction via alignment of subreads; 2) Pre-assembly of long, error-corrected reads; 3) Overlap detection of pre-assembled reads; 4) Overlap filtering; 5) Overlap graph construction; and 6) Graph-based contig construction. After this initial assembly, FALCON-Unzip is used in highly heterozygous species to resolve the distinct haplomes (i.e. to unzip the genome) based on patterns of structural variants and associated SNPs (i.e. haplotype blocks). This unzip process gives rise to a set of so-called primary contigs (the primary assembly) and a set of associated haplotigs (phased variants of the primary contigs, in regions of high heterozygosity). Complete details of the FALCON configuration file used in this study are provided in Additional file 6: Text S3. Finally, the Arrow algorithm within the ‘GenomicConsensus’ PacBio package (https://github.com/PacificBiosciences/GenomicConsensus) was used to polish the phased primary contigs and their associated haplotigs. Genome size was estimated using both k-mer analysis of the error-corrected PacBio reads [75] as well as propidium iodide flow cytometric analysis using *Pisum sativum* L. Citrad (2C = 9.09 pg) as an internal standard (BD Accuri™ C6 Cytometer) [76].

Further polishing and curation of the assembly was accomplished using the Purge Haplotigs pipeline [77]. High levels of heterozygosity in some genomic regions can lead to the incorrect assignment of haplotigs as distinct primary contigs [77]. To identify such errors and correctly assign homologous contigs to the haplotig pool, the Purge Haplotigs pipeline first performs a read-depth analysis using BEDTools [78] to flag abnormally low or high coverage contigs as potential chimeras and then performs a BLAST [79] against the entire assembly to identify putative primary contigs exhibiting high homology to one another. During this process, alignment dotplots are produced, and these are manually screened to break likely chimeras, define the final set of primary contigs as the reference sequence, and assign residual syntenic contigs as haplotigs. Complete details of the Purge Haplotigs process are provided in Additional file 6: Text S4.

### Assessment of genome assembly quality and hi-C scaffolding

Quality of the final curated assembly was assessed using QUAST [80], and assembly completeness was evaluated using the set of 1440 core plant genes in BUSCO v3 [45]. To identify and purge contaminant contigs, the final assembly was BLASTed to the following databases of possible contaminants: plasmid DNA (cpDNA and mtDNA) from angiosperms, the human genome (GRCh38.p7), the *Escherichia coli* genome (CP017100.1), and 16S and 18S rRNAs. The rRNA database was created using the SILVA project [81], and the others were created via sampling from Genbank. To further evaluate completeness, the PacBio error-corrected reads (preads), the RNA-seq data generated for transcriptome assembly (see below), and the GBS data from the BtUCONN1 parent generated for linkage mapping were also aligned to the final assembly using BLASR [82], GMAP [83], and BWA [84], respectively.

To linearly order and orient the primary contigs into chromosome-scale pseudo-molecules, a proximity-guided assembly was performed using Phase Genomics’ Proximo™ chromosome conformation capture (Hi-C) technology [42]. Tissue processing, chromatin isolation, library preparation, sequencing, and Hi-C analysis were performed by Phase Genomics (Seattle, WA, USA). Finally, the BtUCONN1 genetic linkage map was used to manually curate the Hi-C assembly using JuiceBox [85], bringing independent information to guide the ordering of a set of anchor contigs in instances of ambiguity.

### Anchoring of the genetic linkage maps to the physical assembly

Orthogonal sets of markers were used to build the genetic linkage maps of the two parents; thus the two maps share no markers in common, preventing a direct assessment of synteny between the two species. The physical assembly, however, presents a potential “common language” by which the two maps can be compared, provided the markers in the linkage maps can be uniquely located in (i.e. anchored to) the physical assembly. To accomplish this, BLASTn [86] was performed between the MR centroids (queries) and the curated assembly (subject). Using only those centroids exhibiting unique positions within the reference genome, synteny plots were generated using the Pacth function of the Matplotlib plotting library (https://matplotlib.org/index.html). The above anchoring method was also used to project the detected *Pg*-NHR QTL region onto the physical map, thus permitting insight into its underlying physical sequence.

### Transcriptome assembly

For transcriptome assembly, ten different tissues, including immature leaf tissue at various time points after *Pg* inoculation, were collected from a clonally propagated plant of *B. thunbergii* cv. ‘Kobold’ (Additional file 2: Table S3). Fresh tissues were flash frozen in liquid nitrogen and ground to fine powder using mortar and pestle. Total RNA was isolated using the Zymo Research RNA Clean & Concentrator™ kit (Catalog # R1015), according to manufacturers’ protocol. RNAseq libraries were prepared with Illumina TruSeq® RNA Library Prep Kits and sequenced via 150 bp paired-end (PE) reads on an Illumina HiSeq 2500 at the Hubbard Center for Genome Studies, UNH.

CASAVA-processed raw sequences were error-corrected using the software BFC v1.0 [87], following the recommendations of the Oyster River Protocol For Transcriptome Assembly [88]. Error-corrected reads were processed to remove Illumina adapters and gently trimmed to remove low quality reads (Phred ≤5) using Trimmomatic v.0.33 [89]. All post-processed reads from the ten tissues were pooled, and the transcriptome was assembled using Trinity (reference-guided de novo assembly) [34]. Assembly quality was evaluated using TransRate [44], and its completeness was assessed using the set of 1440 core plant genes in BUSCO v3 [45]. In addition to providing basic summary statistics and quality metrics, TransRate provides an overall score of transcriptome contiguity based on a suite of mapping metrics; and BUSCO evaluates assembly content based on the representation of expected single copy orthologs.

### Identification of candidate genes

To facilitate the identification of candidate genes that may explain the association of the detected QTL region to *Pg* response, the physical contigs spanning the QTL region were locally re-assembled using canu [46]. The re-assembled QTL region was then structurally annotated using RepeatMasker [47] and functionally annotated with the Maker pipeline [90], using both ab-initio and transcriptome-based analyses. The set of well-supported genes within the QTL region, hereafter referred to as high-confidence (HC) genes, were defined based on Maker’s Annotation Edit Distance quality metric (AED < 0.7) as well as the requirement that the genes be non-overlapping and between 500 and 10,000 bp in length.

Combinations of approaches were taken to pare down the full set of HC genes to those more likely to contribute to *Pg*-NHR. A differential gene expression (DGE) analysis experiment was conducted to identify genes whose levels of expression detectably change under challenge by *Pg*. Three biological replicates of immature leaves were sampled from clonally propagated *B. thunbergii* cv. ‘Kobold’ plants at four different time points: pre-inoculation (T0) and 48, 72, and 144 h post-inoculation (T48, T72, and T144). Total RNA was extracted, sequenced, and processed as described above. Transcript abundance was quantified using Kallisto [91], and time course analysis was performed using Sleuth [92]. Complete details of the parameters used for transcript abundance and time course analysis are provided in Additional file 6: Text S5.

To complement the above DGE analysis, ~ 428 million PE 100 bp shotgun reads from the *B. vulgaris* parent Wagon Hill (i.e. >30x re-sequencing depth) were aligned to the QTL region in an effort to identify HC genes with no apparent homolog in *B. vulgaris*. The final list of high-priority candidate genes is composed of those HC genes in the QTL region that are either differentially expressed under *Pg* inoculation or have at least one complete CDS sequence absent in *B. vulgaris* (Additional file 2: Figure S5). Putative protein functions and Gene Ontology (GO) terms were assigned to the candidate genes using both the Phytozome v.12.1 [93] and UniProtKB [94] databases.

## Additional files

Additional file 1: Sequencing details of *B. thunbergii* accession ‘BtUCONN1’, *B. vulgaris* accession ‘Wagon Hill’, and the interspecific F1 (*B. ×ottawensis*) mapping population used in this study. (XLSX 20 kb)

Additional file 2: Supplementary Figures and Tables. **Figure S1.** Genetic linkage maps of *B. thunbergii* accession ‘BtUCONN1’ and *B. vulgaris* accession ‘Wagon Hill’. **Figure S2.** Hi-C heat map of the scaffolded primary contigs of *B. thunbergii* cv. ‘Kobold’. **Figure S3**. Venn diagrams of high-priority candidate genes identified for further investigation. **Figure S4.** Time course expression plots for the five candidate genes found via DGE analysis. **Figure S5.** Base-by-base coverage plots in *B. vulgaris* accession ‘Wagon Hill’ for the two candidate genes identified via presence-absence analysis. **Figure S6**. Gel image of the marker used to validate the hybrid status of the individuals in the F_1_ mapping population. **Table S1.** Summary of the raw PacBio data obtained for *B. thunbergii* cv. ‘Kobold’. **Table S2.** Summary statistics of the 14 pseudo-molecules of the* B. thunbergii* cv. 'Kobold' reference assembly. **Table S3.** Details of the library of ten tissues from *B. thunbergii* cv. ‘Kobold’ used for transcriptome assembly. (PDF 5620 kb)

Additional file 3: Linkage map of *B. thunbergii* accession ‘BtUCONN1’ and associated information. (XLSX 412 kb)

Additional file 4: Linkage map of *B. vulgaris* accession ‘Wagon Hill’ and associated information. (XLSX 251 kb)

Additional file 5: MAKER features and detailed functional annotation for the seven candidate genes. (XLSX 13 kb)

Additional file 6: Supplementary Text. **Text S1.** Cluster sequences and primer information for the PCR-based markers used to validate the F_1_ status of the individuals comprising the B. ×*ottawensis* mapping population. **Text S2.** Detailed record of the GBS-SNP-CROP command lines used in this study. **Text S3.** Complete details of the FALCON assembly parameters used in this study. **Text S4.** Complete details of the script used for purging haplotigs. **Text S5.** Complete details of parameters used for quantifying transcripts and the sleuth R code for the time course analysis. (PDF 128 kb)

## Data Availability

All raw sequence data and final assemblies (genome and transcriptome) are available through the NCBI database. The parsed, high-quality GBS data generated for the two parental lines and the 182 F_1_ progeny are available through the NCBI Short Read Archive, with SRA ID’s provided in Additional file 1. RNAseq data from the ten *B. thunbergii* cv. ‘Kobold’ tissues used for DGE and transcriptome assembly are linked to NCBI BioProject PRJNA478022; the assembled transcriptome itself is available under TSA ID GGRA00000000. Data related to the Kobold genome assembly, including the FALCON-Unzip primary contigs and haplotigs, the final Hi-C guided chromosome-level assembly, and all unscaffolded contigs, are deposited in NCBI under BioProject accession number PRJNA478403. The Kobold genome is also available for visualization and annotation through the UNH WebApollo Genome Browser at http://genome.sr.unh.edu/jbrowse.

## Abbreviations

CIM: Composite interval mapping
DGE: Differential gene expression
GBS: Genotyping-by-sequencing
HC genes: High confidence genes
Hpi: Hours post inoculation
LG: Linkage group
MR: Mock reference
NHR: Non-host resistance
PAMP: Pathogen-associated molecular patterns
PE: Paired end
*Pg*: *Puccinia graminis*
SMRT: Single Molecule Real Time
