# Supplementary material for: Mapping non-host resistance to the stem rust pathogen in an interspecific barberry hybrid
Source: BMC Plant Biol. 2019 Jul 16;19:319. doi: 10.1186/s12870-019-1893-9 (PMC6636152; doi:10.1186/s12870-019-1893-9)
Supplement: Supplementary file 2 — Supplementary Figures and Tables. Figure S1. Genetic linkage maps of B. thunbergii accession ‘BtUCONN1’ and B. vulgaris accession ‘Wagon Hill’. Figure S2. Hi-C heat map of the scaffolded primary contigs of B. thunbergii cv. ‘Kobold’. Figure S3. Venn diagrams of high-priority candidate genes identified for further investigation. Figure S4. Time course expression plots for the five candidate genes found via DGE analysis. Figure S5. Base-by-base coverage plots in B. vulgaris accession ‘Wagon Hill’ for the two candidate genes identified via presence-absence analysis. Figure S6. Gel image of the marker used to validate the hybrid status of the individuals in the F1 mapping population. Table S1. Summary of the raw PacBio data obtained for B. thunbergii cv. ‘Kobold’. Table S2. Summary statistics of the 14 pseudo-molecules of the B. thunbergii cv. 'Kobold' reference assembly. Table S3. Details of the library of ten tissues from B. thunbergii cv. ‘Kobold’ used for transcriptome assembly. (PDF 5620 kb) [file 12870_2019_1893_MOESM2_ESM.pdf]

Mapping non-host resistance to the stem rust pathogen in an interspecific barberry hybrid
R Bartaula, A Melo, S Kingan, Y Jin, and I Hale

Additional file 2
Supplementary figures and tables

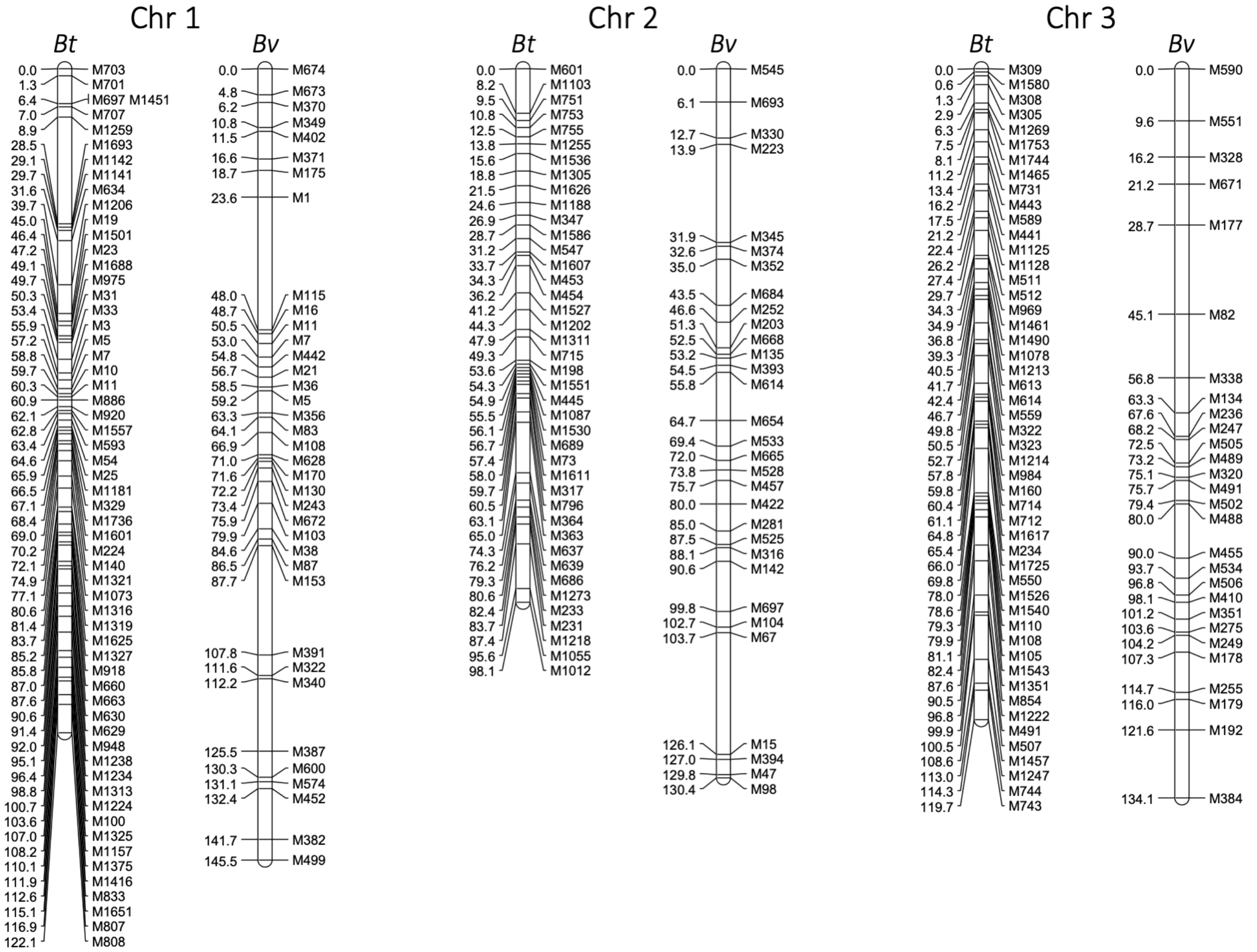

Figure S1 Detailed genetic linkage maps of *B. thunbergii* accession 'BtUCONN1' and *B. vulgaris* accession 'Wagon Hill' (chromosomes 1-3). For each of the 14 chromosomes (paired on the basis of synteny assessed via the Kobold reference assembly), side-by-side ideograms are shown. All chromosomes share the same scale (Kosambi cM) and are oriented such that 0 cM corresponds to the telomere of the short-arm.

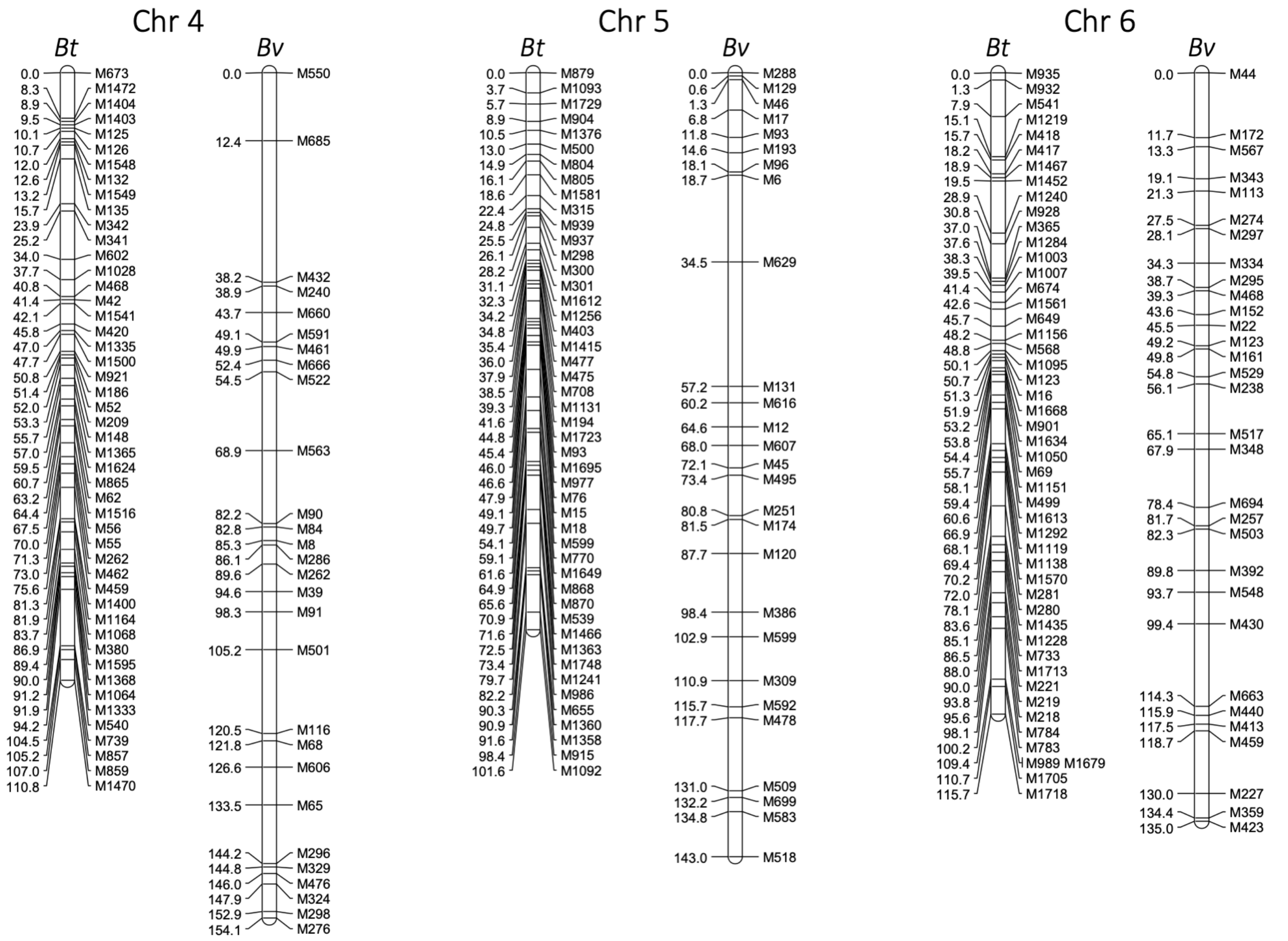

Figure S1 (continued). Detailed genetic linkage maps of *B. thunbergii* accession 'BtUCONN1' and *B. vulgaris* accession 'Wagon Hill' (chromosomes 4-6). For each of the 14 chromosomes (paired on the basis of synteny assessed via the Kobold reference assembly), side-by-side ideograms are shown. All chromosomes share the same scale (Kosambi cM) and are oriented such that 0 cM corresponds to the telomere of the short-arm.

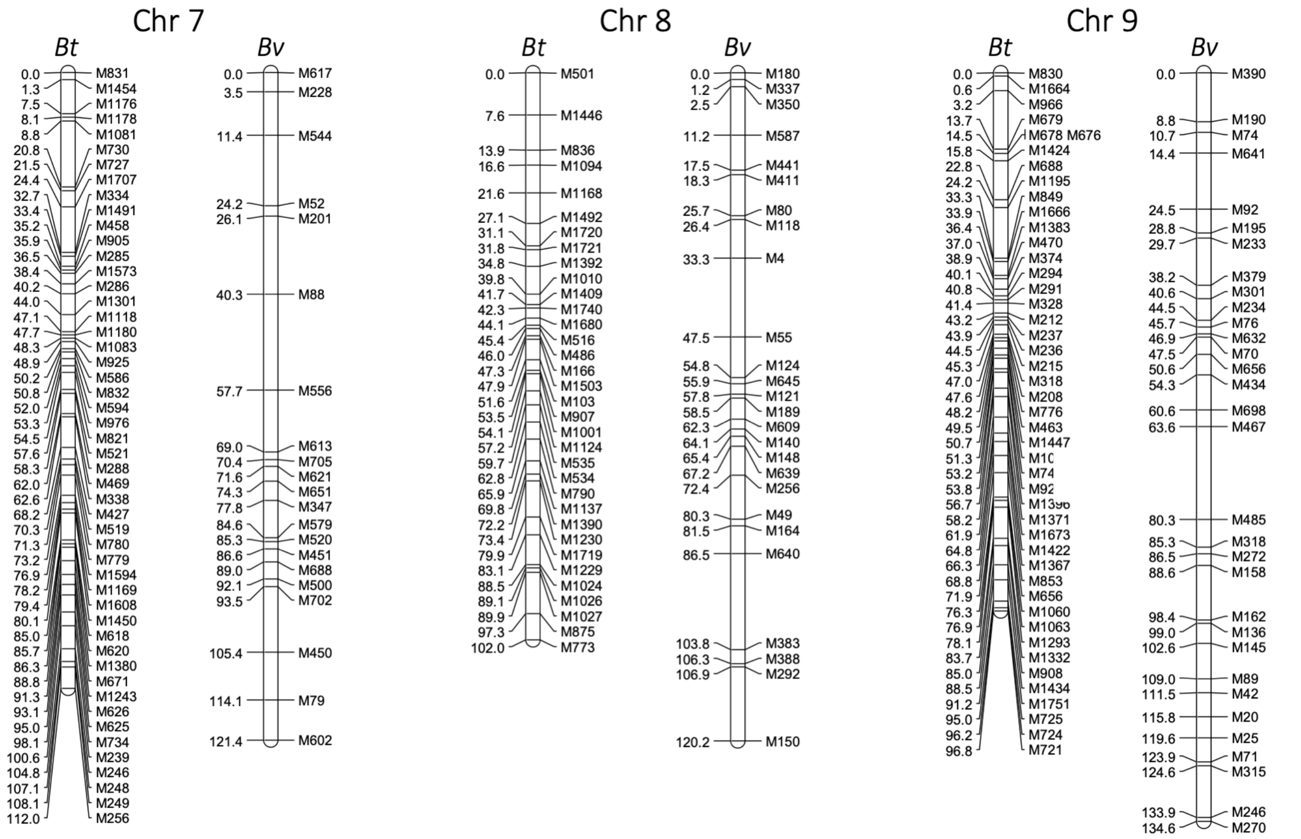

Figure S1 (continued). Detailed genetic linkage maps of *B. thunbergii* accession 'BtUCONN1' and *B. vulgaris* accession 'Wagon Hill' (chromosomes 7-9). For each of the 14 chromosomes (paired on the basis of synteny assessed via the Kobold reference assembly), side-by-side ideograms are shown. All chromosomes share the same scale (Kosambi cM) and are oriented such that 0 cM corresponds to the telomere of the short-arm.

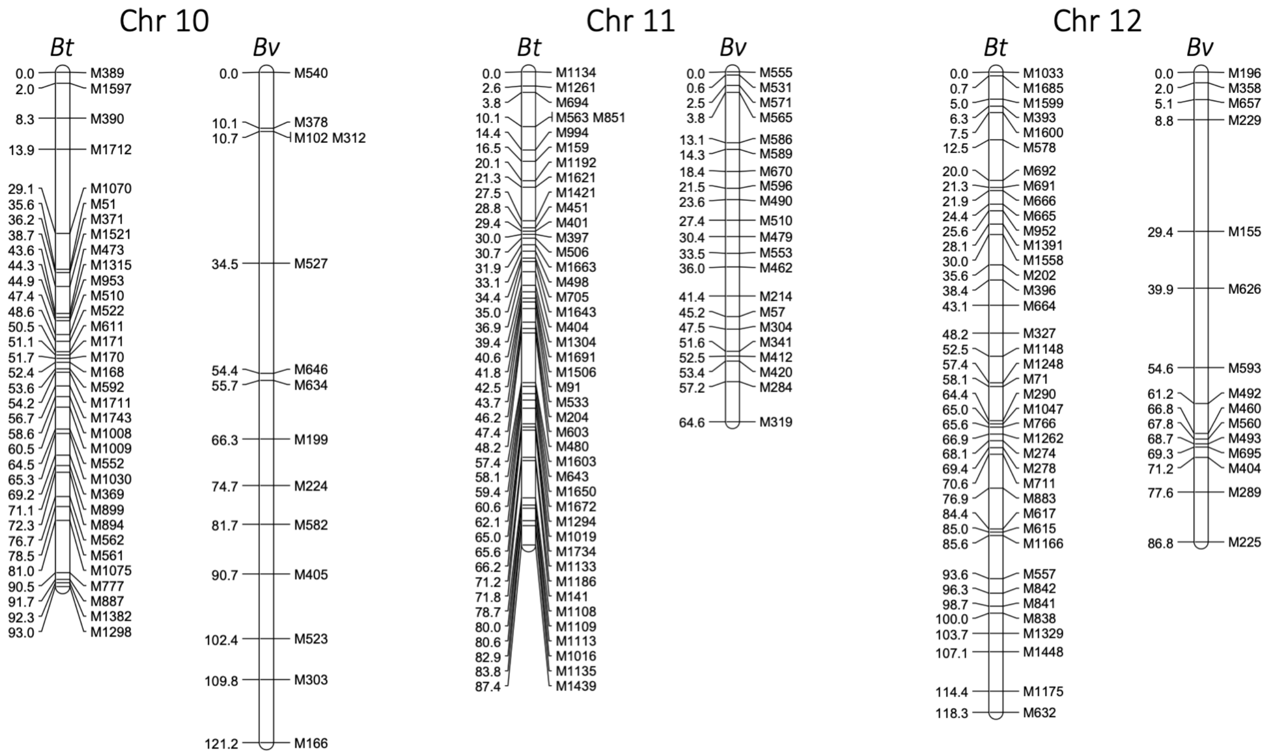

Figure S1 (continued). Detailed genetic linkage maps of *B. thunbergii* accession 'BtUCONN1' and *B. vulgaris* accession 'Wagon Hill' (chromosomes 10-12). For each of the 14 chromosomes (paired on the basis of synteny assessed via the Kobold reference assembly), side-by-side ideograms are shown. All chromosomes share the same scale (Kosambi cM) and are oriented such that 0 cM corresponds to the telomere of the short-arm.

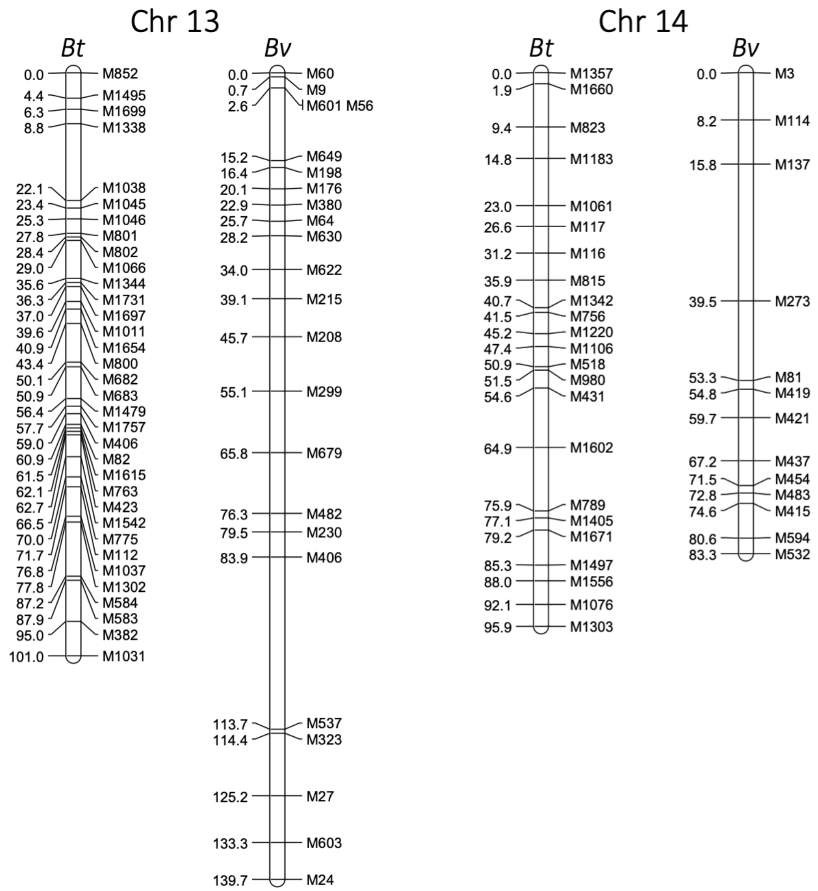

Figure S1 (continued). Detailed genetic linkage maps of *B. thunbergii* accession 'BtUCONN1' and *B. vulgaris* accession 'Wagon Hill' (chromosomes 13-14). For each of the 14 chromosomes (paired on the basis of synteny assessed via the Kobold reference assembly), side-by-side ideograms are shown. All chromosomes share the same scale (Kosambi cM) and are oriented such that 0 cM corresponds to the telomere of the short-arm.

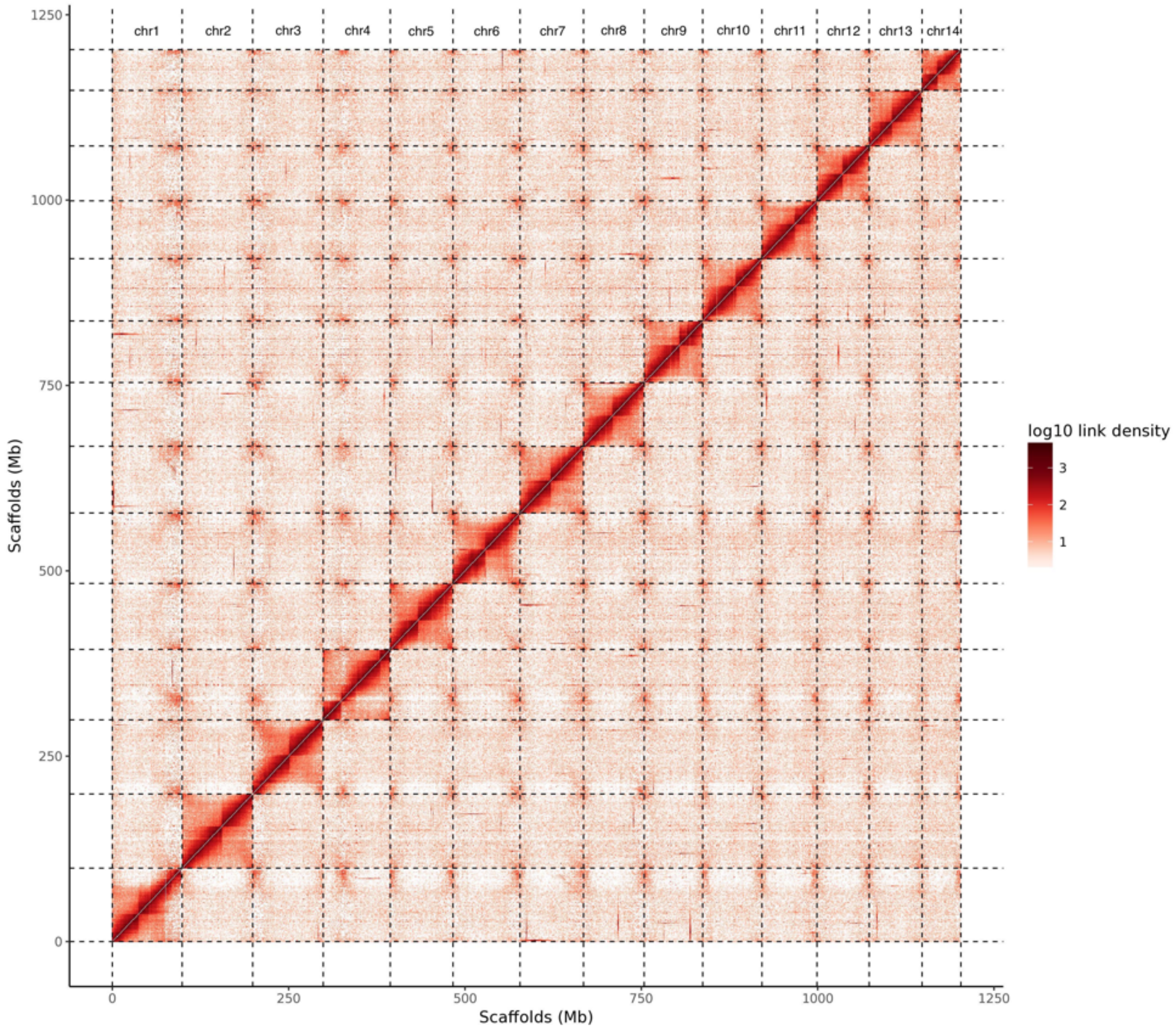

Figure S2. Hi-C post-scaffolding heat map of *B. thunbergii* cv. 'Kobold' primary contigs. Of the 2,698 primary contigs, 97% (2,611 contigs, 1.20 Gbp) successfully assembled into 14 pseudo-molecules representing the 14 chromosomes of *B. thunbergii*. Chromosome designations are made according to physical length, with Chromosome 1 being the longest (99.76 Mbp) and Chromosome 14 being the shortest (54.72 Mbp). See Table S2 for more information.

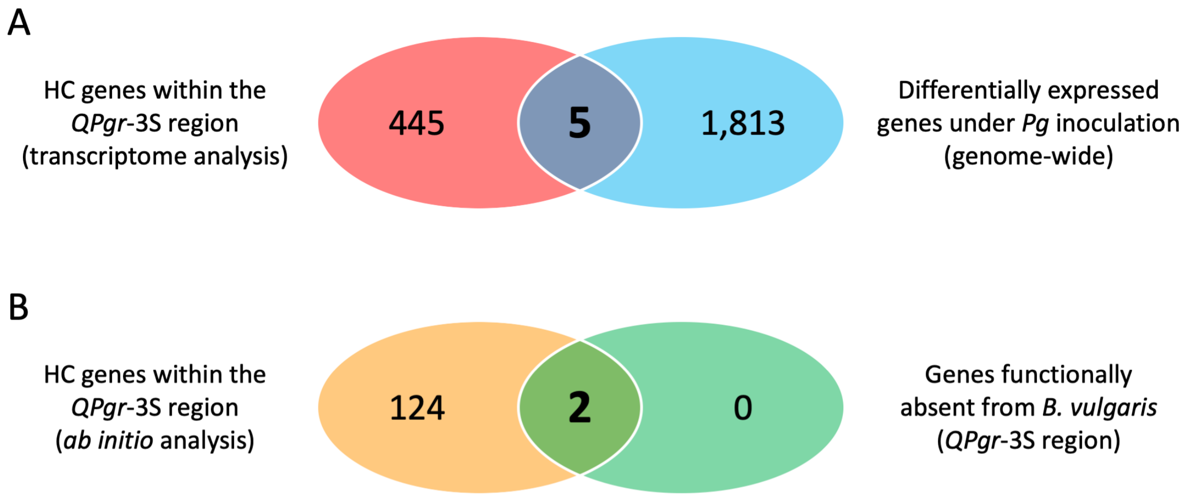

Figure S3. Venn diagrams showing the high-priority candidate genes (intersections) within the QTL region identified for further investigation. Functional annotation of the *QPgr*-3S region resulted in the identification of 576 high confidence (HC) genes, 450 from the transcript-based analysis and 126 based on the *ab-initio* analysis. (**A**) Cross-referencing the 450 transcript-based HC genes with differentially expressed genes under *Pg* inoculation resulted in the identification of five candidates. (**B**) Cross-referencing with genes putatively present in *B. thunbergii* but functionally absent in *B. vulgaris* resulted in the identification of two more.

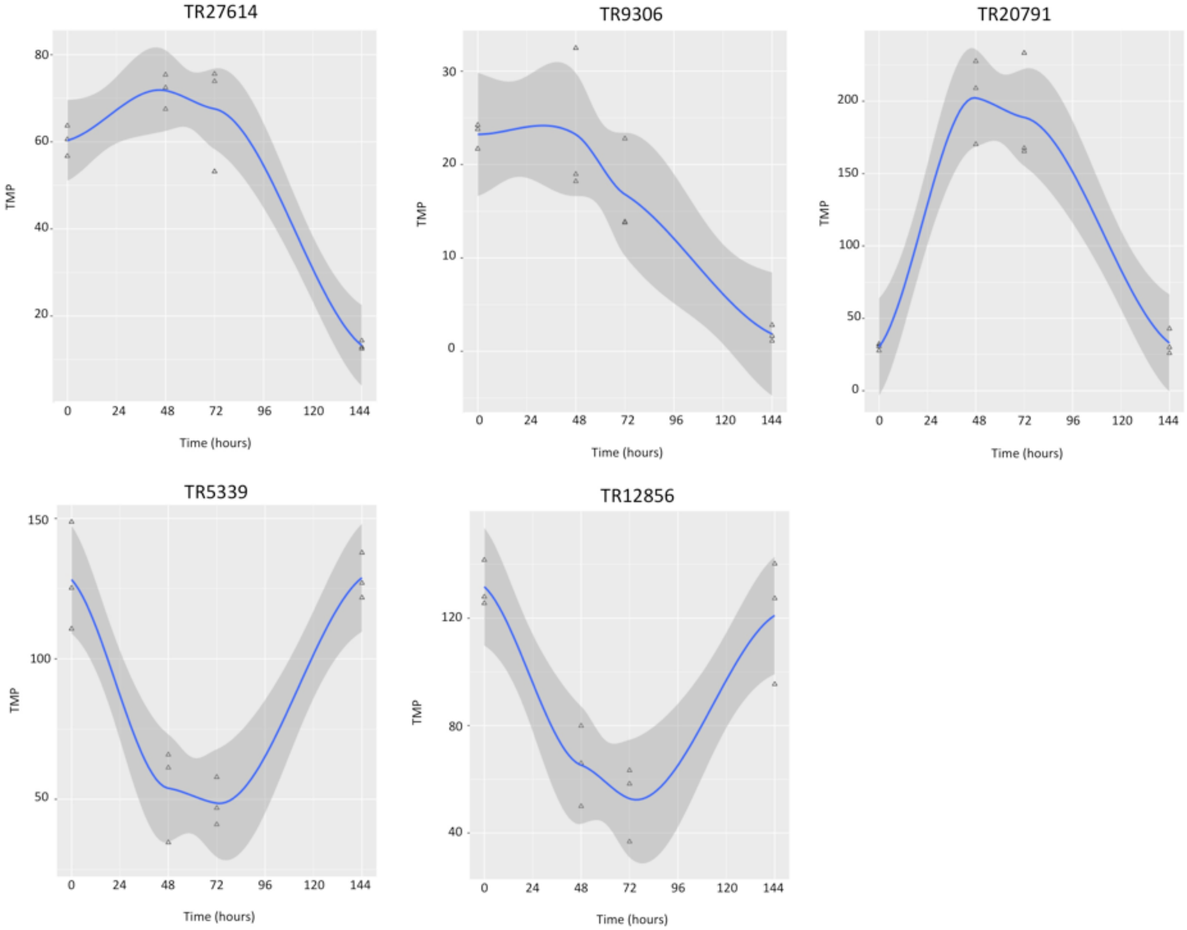

Figure S4. Time course expression plots for the five candidate genes found via DGE analysis.

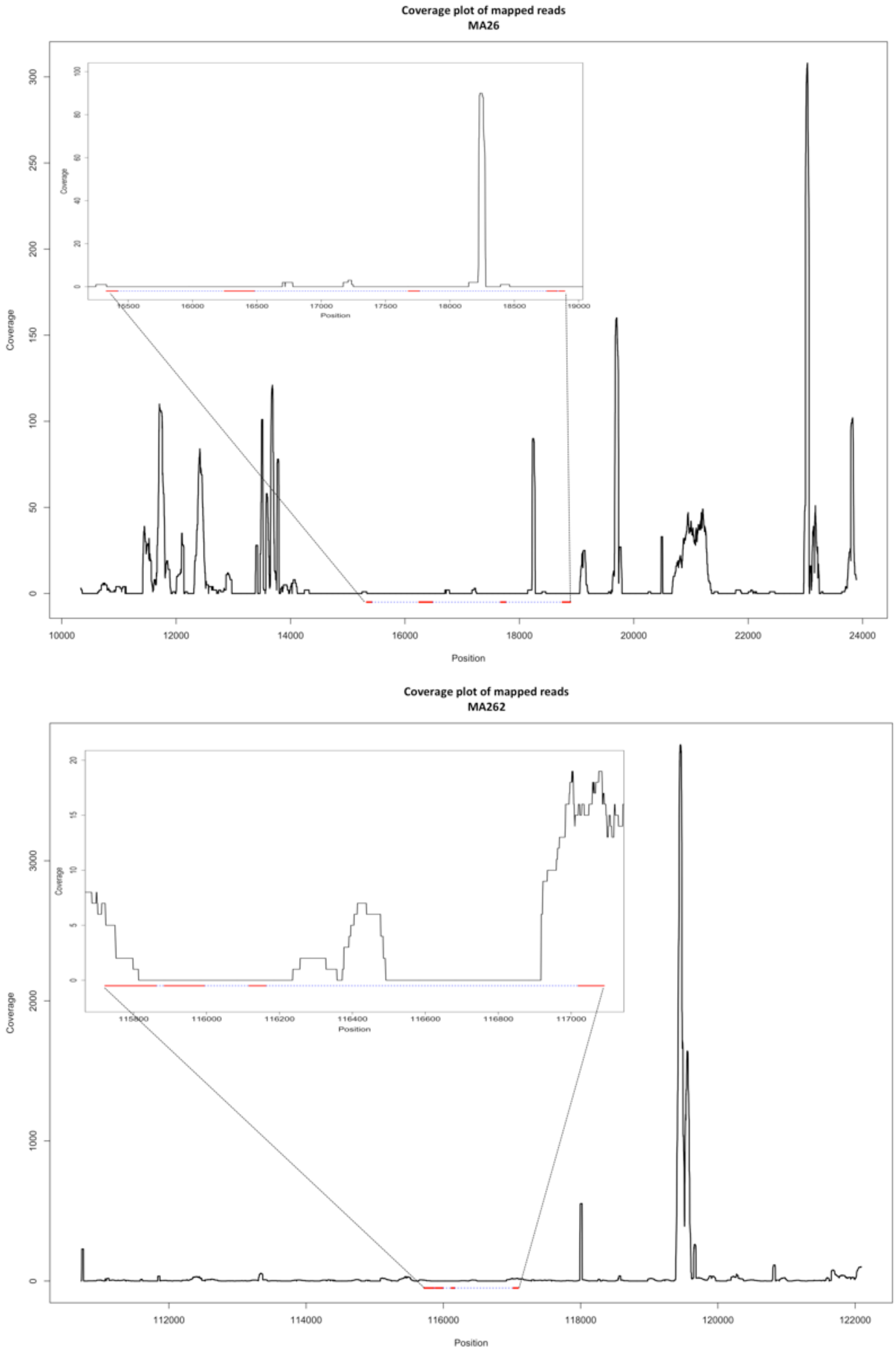

Figure S5. Base-by-base shotgun sequence coverage plots in *B. vulgaris* accession 'Wagon Hill' for the two candidate genes identified via presence-absence analysis. Red solid bars represent the exons spanning the complete CDS sequences within *B. thunbergii* cv. 'Kobold', while the dotted blue lines indicate the interstitial introns. In each of the two panels, the larger plot shows the immediate 10 kbp neighborhood of the gene (gene + 5 kb in each direction) and the inset plot zooms in on the gene sequence itself.

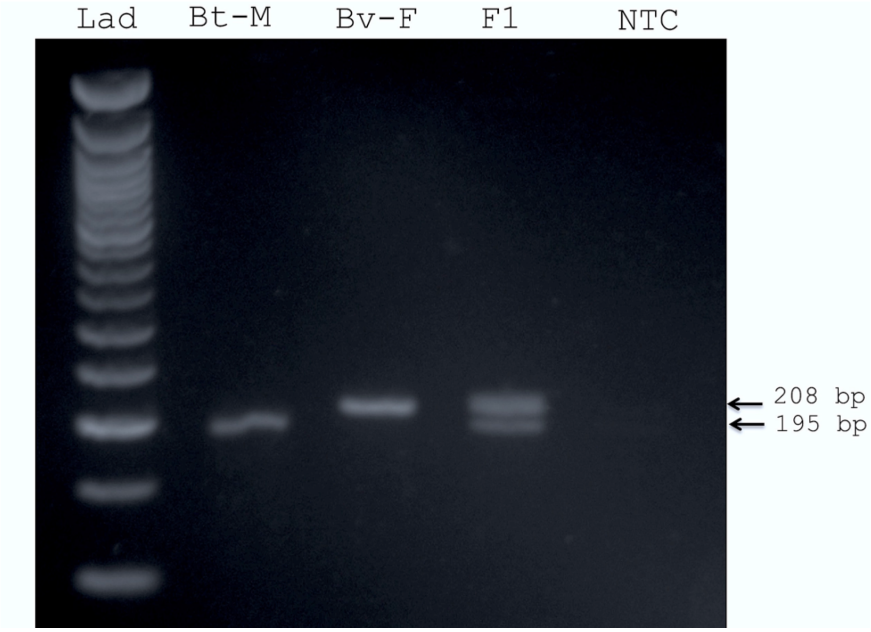

Figure S6. Gel image of the marker used for to validate the hybrid status of the individuals in the F_1 mapping population. The F_1 status of a putative hybrid was considered validated if both bands from the two parental species were detected.

Table S1. Summary of raw PacBio data obtained for *B. thunbergii* cv. 'Kobold' via 115 SMRT cells

Parameter	Value (bp)
Total data generated	128,875,283,508
Number of reads	12,069,440
Median read length	10,630
Mean read length	10,409
N75 read length	18,777
N50 read length	15,021
N25 read length	11,266
Maximum read length	46,433

Table S2. Summary statistics of the 14 pseudo-molecules comprising the *B. thunbergii* cv. 'Kobold' reference assembly

Chr	Length (Mbp)	No. of primary contigs	Contig length N50 (Mbp)	No. of haplotigs	Haplotig length N50 (Mbp)	Total length of haplotigs (Mbp)	Inferred centromere position (Mbp)
Chr 01	99.76	206	0.42	557	0.14	14.20	41.89
Chr 02	99.56	178	5.72	680	0.19	19.97	28.28
Chr 03	97.81	230	0.15	657	0.20	20.62	29.66
Chr 04	94.76	166	1.25	674	0.20	18.53	34.31
Chr 05	94.28	210	0.90	653	0.20	22.10	39.20
Chr 06	89.34	208	3.41	627	0.22	23.04	33.42
Chr 07	88.62	180	2.34	608	0.22	20.31	26.04
Chr 08	85.58	248	2.79	553	0.20	23.03	32.23
Chr 09	83.51	186	1.14	569	0.23	20.43	30.62
Chr 10	82.53	171	1.72	549	0.25	22.58	21.07
Chr 11	77.69	172	0.70	555	0.24	19.63	18.87
Chr 12	74.07	156	1.11	521	0.21	16.30	33.31
Chr 13	73.50	156	1.47	557	0.18	16.69	23.32
Chr 14	54.72	144	1.84	399	0.17	12.31	26.68

Table S3. Details of the library of ten tissues from *B. thunbergii* cv. 'Kobold' used for transcriptome assembly

Tissue ID	Tissue description	Total number of reads (R1 + R2)	Amount of data (Gbp)	Time after *P. graminis* inoculation (hrs)
IM0[a]	Immature leaf	51,672,986	7.75	0
IM48[a]	Immature leaf	54,502,666	8.18	48
IM72[a]	Immature leaf	55,943,898	8.39	72
IM144[a]	Immature leaf	51,843,944	7.78	144
ML	Mature leaf	90,160,600	13.52	--
AM	Apical meristem	20,913,120	3.14	--
YS	Young stem	15,951,214	2.39	--
RO	Root	20,396,332	3.06	--
FR	Fruit	15,367,678	2.31	--
FL	Flowers	20,342,036	3.05	--
Total		397,094,474	59.56	

[a] Immature leaf tissue was collected at four time points, from immediately before *Pg* inoculation up to 144 hours post-inoculation, from rooted clones of the reference *B. thunbergii* cv. 'Kobold'.
