# Supplementary material for: Mapping non-host resistance to the stem rust pathogen in an interspecific barberry hybrid
Source: BMC Plant Biol. 2019 Jul 16;19:319. doi: 10.1186/s12870-019-1893-9 (PMC6636152; doi:10.1186/s12870-019-1893-9)
Supplement: Supplementary file 6 — Supplementary Text. Text S1. Cluster sequences and primer information for the PCR-based markers used to validate the F1 status of the individuals comprising the B. ×ottawensis mapping population. Text S2. Detailed record of the GBS-SNP-CROP command lines used in this study. Text S3. Complete details of the FALCON assembly parameters used in this study. Text S4. Complete details of the script used for purging haplotigs. Text S5. Complete details of parameters used for quantifying transcripts and the sleuth R code for the time course analysis. (PDF 128 kb) [file 12870_2019_1893_MOESM6_ESM.pdf]

Mapping non-host resistance to the stem rust pathogen in an interspecific barberry hybrid

R Bartaula, A Melo, S Kingan, Y Jin, and I Hale

Additional file 6

Supplementary text

Text S1 Cluster sequences and primer information for PCR-based markers used for validating the F1 status of the putative hybrid individuals comprising the *B. ×ottawensis* mapping population. Primer sites are underlined, and the 13 bp indel sequence is highlighted in green.

```
>B.thunbergii_Cluster45383_222bp
AATAATAGCTCCCTGATTGGGGCTCATTATCAGTCGCCATGTTTAGAATCCCGAGCAAGAAGCTCGGGAAAAATCAGAA
GCACATGCAATAGATAGAAGAAGCAAAGAGGTATTCTTCAAAGCTCCAAATCCCCTAAAAAAAACGTGGTCGGTTCTGC
TCGGCTAGGTTGCTTCAAATATAGCATTTCAGCTCGGAATTCCTCACTTTTCTTCTCAGCTCCG

>B.vulgaris_Cluster45383_235bp
AATAATAGCTCCCTGATTGGGGCTCATTATCAGTCGCCATGTTTAGAATCCCGAGCAAGAAGCTCGGGAAAAATCAGAA
GCACATGCAATAGATAGAAGAAGCAAAGAGATCGATCAAAGAGGTATTCTTCAAAGCTCCAAATCCCCTAAAAAAAACG
TGGTCGGTTCTGCTCGGCTAGGTTGCTTCAAATATAGCATTTCAGCTCGGAATTCCTCACTTTTCTTCTCAGCTCCG
```

```
Bt      AATAATAGCTCCCTGATTGGGGCTCATTATCAGTCGCCATGTTTAGAATCCCGAGCAAGA     60
Bv      ............................................................

Bt      AGCTCGGGAAAAATCAGAAGCACATGCAATAGATAGAAGAAGCAAAGAG-----------     120
Bv      .................................................ATCGATCAAAG

Bt      --GTATTCTTCAAAGCTCCAAATCCCCTAAAAAAAACGTGGTCGGTTCTGCTCGGCTAGG     180
Bv      AG..........................................................

Bt      TTGCTTCAAATATAGCATTTCAGCTCGGAATTCCTCACTTTTCTTCTCAGCTCCG          235
Bv      .......................................................
```

Species	Primer ID	Primer sequence	Annealing Tm	Band size (bp)
B. thunbergii	Cluster45383_UF	CCTGATTGGGGCTCATTATC	52 ºC	195
B. vulgaris	Cluster45383_UR	AGTGAGGAATTCCGAGCTGA		208

Text S2 Detailed record of the GBS-SNP-CROP v.3.0 command lines used in this study, including all specified pipeline parameters [1]

```
# GBS-SNP-CROP-1.pl
perl GBS-SNP-CROP-1.pl -d PE -b barcodesIDs.txt -fq L001 -s 1 -e
48 -enz1 TGCA -enz2 CGG

# GBS-SNP-CROP-2.pl
perl GBS-SNP-CROP-2.pl -d PE -fq L001 -t 10 -ph 33 -ad TruSeq3-
PE.fa:2:30:10 -l 30 -sl 4:30 -tr 30 -m 32

# GBS-SNP-CROP-3.pl
perl GBS-SNP-CROP-3.pl -d PE -b barcodesIDs.txt -fq L001

# GBS-SNP-CROP-4.pl
perl GBS-SNP-CROP-4.pl -d PE -b barcodeID.txt -rl 150 -pl 32 -p
0.01 -id 0.93 -t 20 -MR ParentsMR

# GBS-SNP-CROP-5.pl
perl GBS-SNP-CROP-5.pl -d PE -b barcodeID.txt -ref
ParentsMR.MockRef.Genome.fasta -Q 30 -q 0 -f 2 -F 2308 -t 10 -Opt
0

# GBS-SNP-CROP-6.pl
perl GBS-SNP-CROP-6.pl -b barcodeID.txt -out SNPs.summary.txt -
indel -t 20

# GBS-SNP-CROP-7.pl
perl GBS-SNP-CROP-7.pl -in SNPs.summary.txt -out SNPs.call.txt -
indel -mnHoDepth0 5 -mnHoDepth1 20 -mnHetDepth 3 -altStrength
0.962 -mnAlleleRatio 0.25 -mnCall 0.75 -mnAvgDepth 3 -mxAvgDepth
200

# GBS-SNP-CROP-8.pl
perl GBS-SNP-CROP-8.pl -in SNPs.call.txt -out SNP.Rmatrix -b
barcodesIDs.txt -formats R
```

Text S3 Complete details of the FALCON [2] assembly parameters used in this study

```
[General]
# list of files of the initial fasta
input_fofn = input.fofn

input_type = raw
#input_type = preads

#openending = True

stop_all_jobs_on_failure = False

# The length cutoff used for seed reads used for initial mapping
length_cutoff = 5000

genome_size = 1400000000
seed_coverage = 30

# The length cutoff used for seed reads usef for pre-assembly
length_cutoff_pr = 9000


sge_option_da = -pe smp 5 -q bigmem
sge_option_la = -pe smp 20 -q bigmem
sge_option_pda = -pe smp 6 -q bigmem
sge_option_pla = -pe smp 16 -q bigmem
sge_option_fc = -pe smp 24 -q bigmem
sge_option_cns = -pe smp 12 -q bigmem

pa_concurrent_jobs = 96
cns_concurrent_jobs = 96
ovlp_concurrent_jobs = 96


pa_HPCdaligner_option =  -v -B128 -M32 -e.70 -l4800 -s100 -k18 -
h480 -w8
ovlp_HPCdaligner_option = -v -B128 -M32 -h1024 -e.96 -l2400 -s100
-k18


pa_DBsplit_option = -a -x500 -s400

ovlp_DBsplit_option = -s400


falcon_sense_option = --output_multi --min_idt 0.70 --min_cov 2 -
-max_n_read 200 --n_core 8
falcon_sense_skip_contained = True

overlap_filtering_setting = --max_diff 85 --max_cov 87 --min_cov
2 --n_core 12
```

Text S4 Complete details of the script used for purging haplotigs

Purge Haplotigs version from April 15th, 2017 [3]

https://bitbucket.org/mroachawri/purge_haplotigs/src/f6b0eea4975e534ea4e9ef29e5c9fb22b0782d5f

```
$ zz0_coverage_histogram.sh aligned.sorted.bam
```

A BAM file was generated in the SMRTLink resequencing pipeline v3.2.0 by aligning all subreads to the FALCON-Unzip primary contigs using BLASR (5.3.98a8d00) [4]

```
$ zz1_analyse_gencov.pl  -i genecov.out  -o stats.csv  -l 11  -m 48  -h 75
```

```
$ zz2_assign_contigs.sh  stats.csv  genome.fasta
```

```
$ zz3_reassign_contigs.pl  -t suspect_contig_reassign.tsv  -g genome.fasta  -o output_prefix
```

The default of 3 rounds of purging was run.

Text S5 Complete details of parameters used for quantifying transcript and the sleuth R code for the time course analysis

Kallisto [5] command lines for quantifying transcript abundance. The quant function was used for all seven RNA-seq libraries:

```
$ kallisto index -i Ber_ALL7.idx ALL7.TrinityGG.Assembly.fa
$ kallisto quant -t 20 -i Ber_ALL7.idx -o IM0_R1_KOUT \
/path/to/dir/IM0-1_PE_R1.fq
/path/to/dir/IM0-1_PE_R1.fq
$ kallisto quant -t 20 -i Ber_ALL7.idx -o IM0_R2_KOUT \
/path/to/dir/IM0-2_PE_R1.fq
/path/to/dir/IM0-2_PE_R1.fq
$ kallisto quant -t 20 -i Ber_ALL7.idx -o IM0_R3_KOUT \
/path/to/dir/IM0-3_PE_R1.fq
/path/to/dir/IM0-3_PE_R1.fq
```

Sleuth [6] R command lines for time course analysis:

```
library("sleuth")
sample_id = dir(file.path(".", "kallistoOUT"))
kal_dirs = file.path(".", "KallistoOUT", sample_id, "kallisto")
s2c = read.table(file.path(".", "hiseq_info.txt"), header = T,
stringsAsFactors=F)
s2c = dplyr::select(s2c, sample = sample, condition)
s2c = dplyr::mutate(s2c, path = kal_dirs)
so = sleuth_prep(s2c,
extra_bootstrap_summary=T,read_bootstrap_tpm=T)
so = sleuth_fit(so, ~condition, 'full')
so = sleuth_fit(so, ~1, 'reduced')
so = sleuth_lrt(so, 'reduced', 'full')
sleuth_table = sleuth_results(so, 'reduced:full', 'lrt', show_all
= F)
sleuth_significant = dplyr::filter(sleuth_table, qval <= 0.01)
wtest = sleuth_wt(so, 'conditionTime0', 'full')
swbeta = sleuth_lrt(wtest, 'reduced',  'full')
sleuth_live(swbeta)
```

References

1. Melo AT, Bartaula R, Hale I. GBS-SNP-CROP: a reference-optional pipeline for SNP discovery and plant germplasm characterization using variable length, paired-end genotyping-by-sequencing data. BMC Bioinformatics. 2016;17:29.

2. Chin C-S, Peluso P, Sedlazeck FJ, Nattestad M, Concepcion GT, Clum A, et al. Phased diploid genome assembly with single-molecule real-time sequencing. Nat Methods. 2016;13:1050.

3. Roach MJ, Schmidt SA, Borneman AR. Purge Haplotigs: allelic contig reassignment for third-gen diploid genome assemblies. BMC Bioinformatics. 2018;19:460.

4. Chaisson MJ, Tesler G. Mapping single molecule sequencing reads using basic local alignment with successive refinement (BLASR): application and theory. BMC Bioinformatics. 2012;13:238.

5. Bray NL, Pimentel H, Melsted P, Pachter L. Near-optimal probabilistic RNA-seq quantification. Nat Biotechnol. 2016;34:525.

6. Pimentel H, Bray NL, Puente S, Melsted P, Pachter L. Differential analysis of RNA-seq incorporating quantification uncertainty. Nat Methods. 2017;14:687.
